# **Fiber-specific differences in protein content of pathways related to mTORC1 signaling and oxidative metabolism in individuals with obesity**

**DOI:** 10.1038/s41598-025-09169-7

**Published:** 2025-07-04

**Authors:** Cristian Campos, Marcelo Flores-Opazo, Denisse Valladares-Ide, Claudio Cabello-Verrugio, Pilar Parada, Francisco Morales, Joseline Arredondo, Luis Peñailillo

**Affiliations:** 1 Exercise and Rehabilitation Sciences Institute, Faculty of Rehabilitation Sciences, School of Physical Therapy, Universidad Andres Bello, Santiago, Chile; 2 Long Active Life Laboratory, Instituto de Ciencias de la Salud, Universidad de O’Higgins, Rancagua, Chile; 3 Center for Research on Pandemic Resilience, Faculty of Life Sciences and Institute of Public Health, Universidad Andres Bello, Santiago, Chile; 4 Laboratory of Muscle Pathology, Fragility and Aging, Faculty of Life Sciences, Universidad Andres Bello, Santiago, Chile; 5 Centro de Biotecnología de Sistemas, Universidad Andres Bello, Santiago, Chile; 6 Especialidad en Medicina del Deporte y la Actividad Física, Facultad de Ciencias Médicas, Universidad De Santiago, Santiago, Chile; 7 School of Physical Therapy, Universidad Andres Bello, 700 Fernandez Concha, Las Condes, Santiago 7591538 Chile

**Keywords:** Skeletal muscle, Muscle ubiquitination, Muscle protein synthesis, Mitochondrial complex, Overweight, Obesity, Energy metabolism

## Abstract

This study aimed to compare the protein content of pathways related to mTORC1 signaling and oxidative metabolism in whole skeletal muscle and isolated muscle fibers from healthy individuals (HEALTHY) and individuals with obesity (OBESE). Muscle biopsies were obtained from 18 individuals. Samples were freeze-dried, and fibers were isolated and fiber-typed for type I and IIa myosin heavy chain isoforms. The protein content of Akt-1, mTOR, p70s6K, S6 ribosomal protein (S6RP), ERK1/2, p38, total protein ubiquitination (TPU), and OXPHOS proteins in whole muscle and isolated fibers was determined using immunoblotting. Muscle fiber morphology was also assessed. Fiber type proportion was similar in HEALTHY, while OBESE showed a greater proportion of type IIa fibers. Fiber cross-sectional area (CSA) was similar in HEALTHY, while OBESE showed greater CSA of type I fibers. Protein content of p70s6K was lower (73%, *p* = 0.008), while ERK1/2 (29%, *p* = 0.05) and TPU (32%, *p* = 0.03) were increased in whole muscle from OBESE compared with HEALTHY. Fiber-specific differences were found in p38 (IIa > I, *p* = 0.03) in HEALTHY, while OBESE showed different complex II and V (I > IIa, *p* = 0.03 and *p* = 0.04, respectively) between fibers. Furthermore, a negative correlation was found between body mass and p70s6K protein content. Our findings suggest that obesity impairs the mTORC1-related protein pathway while increasing degradation in individuals with obesity.

**Supplementary Information:**

The online version contains supplementary material available at 10.1038/s41598-025-09169-7.

## Introduction

Obesity is linked to chronic diseases such as type 2 diabetes, cardiovascular diseases, and cancer^1,2^. Obesity also leads to muscle dysfunction, characterized by reduced muscle function due to metabolic impairment, sedentary behavior, adipose tissue, comorbidities, and inflammation^3,4^. Obesity affects protein synthesis, degradation, and substrate oxidation in skeletal muscle^5^, and as skeletal muscle accounts for 40–50% of body mass, these changes can disrupt whole-body metabolism and overall health^6^. Protein synthesis is majorly regulated by the activity of the mTORC1-related proteins pathway (Akt/mTOR/p70s6K/S6K), which responds to various stimuli such as mechanical loading, growth factors, and nutrition^7^. Sullivan et al. found that individuals with obesity have lower levels of insulin-like growth factor-1 (IGF-1) in skeletal muscle compared to lean individuals, which may contribute to reduced protein synthesis in skeletal muscle^8^. However, muscle mass is also regulated by its degradation, which is primarily controlled by the ubiquitin-proteasome system^9^, especially during muscle atrophy, where protein degradation exceeds protein synthesis^9^. Obesity induces an aberration of circulating factors (e.g., insulin, growth hormones, IGF-1, androgens, and pro-inflammatory cytokines), which, in addition to reduced physical activity, impact protein synthesis and degradation^10^. Furthermore, impaired function of oxidative metabolism proteins (i.e., mitochondrial proteins) has also been reported in obesity, which could impair muscle metabolism^10^.

Skeletal muscle consists of a heterogeneous composition of muscle fibers, including types I, IIa, and IIx in humans^11,12^. The myofibrillar protein pool, comprising the various isoforms of the protein myosin heavy chain (MHC), accounts for approximately half of the total protein in muscle. MCH isoforms (MHC-I, MHC-IIa, and MHC-IIx) provide a marker to characterize muscle fibers^13^. These fibers differ in energy metabolism, force production, contraction speed, and fatigue resistance^14^, and the proportion of these fibers in skeletal muscle determines the metabolic responses and function of a given muscle^15^. Due to these functional differences, muscle fibers may adapt differently to daily activity, training types, and nutritional stimuli^16,17^. Between-group comparisons revealed greater prevalence of fast muscle fibers (type II) and lower prevalence of slow fibers (type I) in individuals with obesity compared to lean individuals^15,18^. Furthermore, a lower proportion of type I fibers has been associated with an increased risk of obesity^15^. Thus, as the proportion of fiber types differs among individuals with obesity, it is also possible that differential adaptations (i.e., key metabolic protein content) to environmental factors (e.g., nutrition and physical activity) of each fiber type may vary in individuals with obesity. For example, differential fiber-type specific protein expression in response to exercise has been described^19^. The protein synthesis and degradation processes seem to be tightly regulated in a fiber-specific manner^20^ and may be dysregulated in obesity^21^. However, the exact mechanisms remain unclear^22^. Nowadays, fiber-specific analyses can be implemented to determine differences across fiber types, which could better represent adaptations within skeletal muscle. This study aimed to explore potential differences in protein content levels of key proteins involved in protein synthesis and degradation, as well as oxidative metabolism-related pathways, in type I and IIa skeletal muscle fibers and whole muscle homogenates in obese and healthy individuals. We hypothesized that individuals with obesity exhibit lower levels of mTORC1-related proteins and impaired oxidative metabolism pathways, which show fiber-specific adaptations.

## Methods

### Participants

Eighteen participants were included in this study. Nine individuals with obesity and nine healthy individuals were recruited from the university surroundings, whose physical characteristics are presented in Table 1. All individuals reported no musculoskeletal or neuromuscular pathologies or previous muscle damage in the lower extremities. The Ethics Committee of the Pontificia Universidad Católica de Chile approved this study (N°01, 07-01-2021, Date: 7 January 2022), and all participants signed the Informed Consent form, following the principles outlined in the Helsinki Declaration. Participant recruitment began on February 1, 2022.

**Table 1:** Participants’ physical characteristics (mean ± SD).

	Obese	Healthy	*P*-value
Men/Women	4/5	6/3	
Age (years)	41.2 ± 7.6	30.4 ± 6.9	0.01
Height (cm)	167.9 ± 9.9	167.9 ± 8.1	0.99
Body mass (kg)	96.3 ± 12.7	70.2 ± 6.8	0.001
Body mass index (kg/m^2^)	34.3 ± 4.2	24.8 ± 1.5	0.001
Physical activity level (METS-min/week)	1,720 ± 1,834.2	3,011.1 ± 1,828.6	0.08

### Muscle samples

Participants attended the laboratory after overnight fasting without exercising in the previous 48 h. Body mass, height, and physical activity level (PAL) were recorded before any procedure in all individuals. Muscle samples were obtained from the vastus lateralis (VL) using the modified Bergstrom´s needle technique with suction, as previously described^19^. Briefly, muscle samples were separated from fatty tissue and blood, and then divided into different portions for various analyses. Approximately 10 mg of muscle mass was kept for fiber type isolation, while 20 mg was kept for whole muscle protein analyses by Western blot and histology analyses. All samples were snap-frozen in liquid nitrogen and stored at -80 °C until analysis.

### Fiber type isolation

Muscle samples (~ 5 mg) were freeze-dried for 48 h. After lyophilization, fibers were manually separated under a light microscope using forceps and collected into a 10 µl loading buffer. 30–60 single fiber segments were collected from each sample. Muscle fiber types were identified by dot blotting and grouped according to the protocol described in a previous study^23^.

The dot blot procedure is shown in Supplementary Fig. 1. In brief, samples were spotted on two separate PVDF membranes for the identification of type I and type IIa fibers. After absorption, the membranes were reactivated and equilibrated, then incubated with either MHC IIa (mouse monoclonal IgG, clone A4.74, Developmental Studies Hybridoma Bank [DSHB]) or MHC I (mouse monoclonal IgM, clone A4.840, Developmental Studies Hybridoma Bank [DSHB]) antibodies overnight at 4 °C. Following washing, the membranes were treated with a secondary antibody. Immunoreactivity detection was performed using a chemiluminescent substrate. Fibers showing positivity for both types were excluded from the analysis. Type IIx fibers were also identified; however, as established, pools must contain a minimum of nine fibers to be considered representative^23^. This threshold was only met in four individuals; hence, type IIx fibers were excluded from the analysis in the present study. On average, we isolated 29.4 ± 12.6 type I fibers, 31.3 ± 16.9 type IIa fibers, and 7.3 ± 6.8 assumed hybrid fibers were discarded per subject. In total, we isolated 1,288 fibers, from which 560 were type I, 596 were type IIa, and 132 were discarded as assumed to be hybrid fibers.

### Western blot

Total protein content from whole muscle homogenates and isolated pooled fibers were separated in 10% TGX Stain-free Fast-Cast Acrylamide gels (Bio-Rad, Hercules, CA, USA) and transferred to Nitrocellulose membranes (Thermo Scientific, Rockford, IL, USA). Membranes were blocked and then incubated overnight with a primary antibody. Full details of antibodies utilized in these analyses are presented in Table 2 (Supplementary material). Rabbit anti mTOR^24^, Akt-1^25^, p70s6K^26^, Ubiquitin^27^, ERK1/2^28^, p38^29^ and mouse anti S6RP^26^ antibodies were from Cell Signaling. Mouse anti OXPHOS^30^ antibody was from Invitrogen. β-tubulin^31^ and MCH^32^ were used as loading controls to normalize the results obtained from whole muscle and specific fibers, respectively^33,34^. Membranes were incubated with the proper secondary antibody at room temperature for 1.5 h. The immunoreactive detection was made with SuperSignal™ West Pico PLUS Chemiluminescent Substrate (Thermo Scientific, Rockford, IL, USA).

Glial fibrillar acidic protein (GFAP) and vimentin proteins were evaluated to compare the presence of other cell types remaining in the pool of specific fibers after isolation between whole muscle homogenates and isolated fibers. GFAP is present in nervous tissue as a motor neuron^35^, and vimentin^36^ is an intermediate filament in all cell types except skeletal muscle^37^.

### Immunofluorescence

Serial Cryosections (10–14 μm thick) were fixed and blocked with PBS containing 2% BSA for 1 h. Specific primary antibodies were incubated overnight in the same blocking buffer at 4° C. Antibodies directed against slow and fast IIa myosin heavy chains were the same as for dot blot. Representative images were acquired with a Motic BA310 fluorescence microscope (Motic, Hong Kong) and analyzed with ImageJ v1.54i (NIH, USA) for fiber type and size quantification.

### Statistical analysis

All variables passed the Shapiro-Wilk normality test. Participants’ physical characteristics were compared using an independent Student´s t-test. An independent Student´s t-test was used to compare whole muscle homogenates between the Obese and Healthy groups for all proteins analyzed. A two-way repeated measures ANOVA was used to compare changes in muscle protein content of type I and IIa muscle fibers between the Obese and Healthy groups. If a significant main effect was found, pairwise comparisons were made with Sidak’s post hoc test. The significance level was set at *P* < 0.05. All statistical analyses were performed with Prism 9.0 (GraphPad, USA). An independent Student´s t-test was used to compare vimentin and GFAP protein contents between whole muscle homogenates and isolated muscle fibers. We performed a Pearson´s correlation coefficient analysis between body mass and BMI vs. all muscle variables. Data are presented as mean ± standard deviation (SD).

## Results

### Participants physical characteristics

Table 1 shows that individuals with obesity exhibit a greater BMI and body mass compared with healthy individuals. However, the obese group was statistically older than the healthy individuals. PAL was statistically similar, with a tendency to greater PAL in the healthy group.

### Changes in size and proportion of muscle fibers

**Fig. 1:**
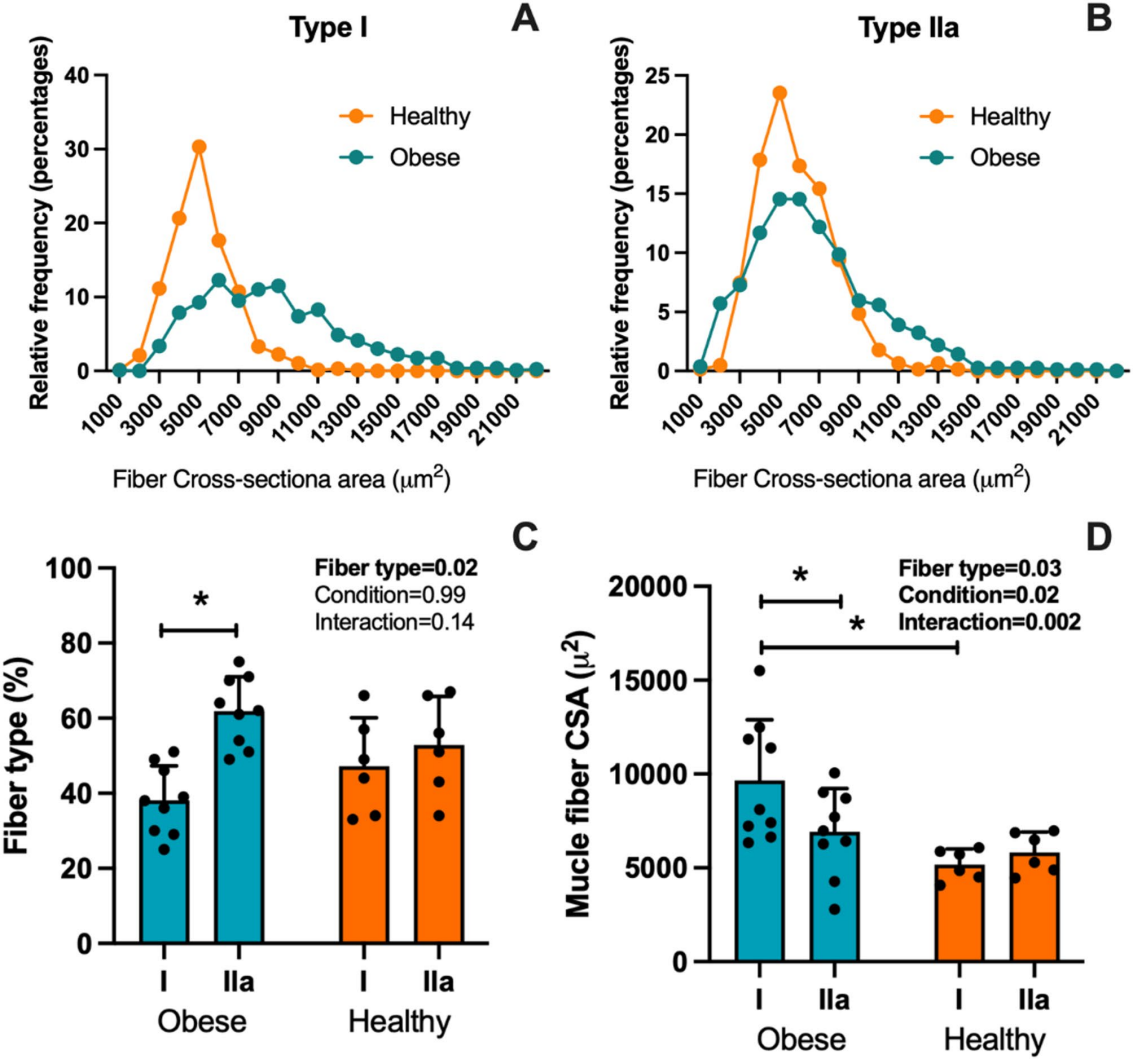
The histogram shows the distribution of cross-sectional area of type I and IIa muscle fibers from Healthy (orange) and Obese (green) individuals (**A**-**B**). Proportion of type I and IIa muscle fibers (as % of the total number of fibers) (**C**) and cross-sectional area (CSA) of muscle fibers (**D**). * : *P* < 0.05.

Figure 1A and B present the histogram of the CSA of type I and IIa muscle fibers of obese and healthy individuals. Figure 1C shows that obese individuals showed a greater proportion of type IIa fibers than type I (61.8 ± 9.1% vs. 38.1 ± 9.2%; *P* = 0.01), while healthy individuals showed a similar proportion between type I and IIa fibers (47.2 ± 12.9% vs. 52.8 ± 12.9%; *P* = 0.78). The fiber CSA of obese individuals showed greater (*P* = 0.0006) CSA of type I (9,664.7 ± 3,231.5 µm^2^) than type IIa (6,918.7 ± 2,314.3 µm^2^) fibers, while CSA of healthy individuals was similar (*P* = 0.59) in type I (5,182.8 ± 818.0 µm^2^) and IIa (5,828.3 ± 1,083.2 µm^2^) fibers (Fig. 1D).

### mTORC1-related proteins

**Fig. 2:**
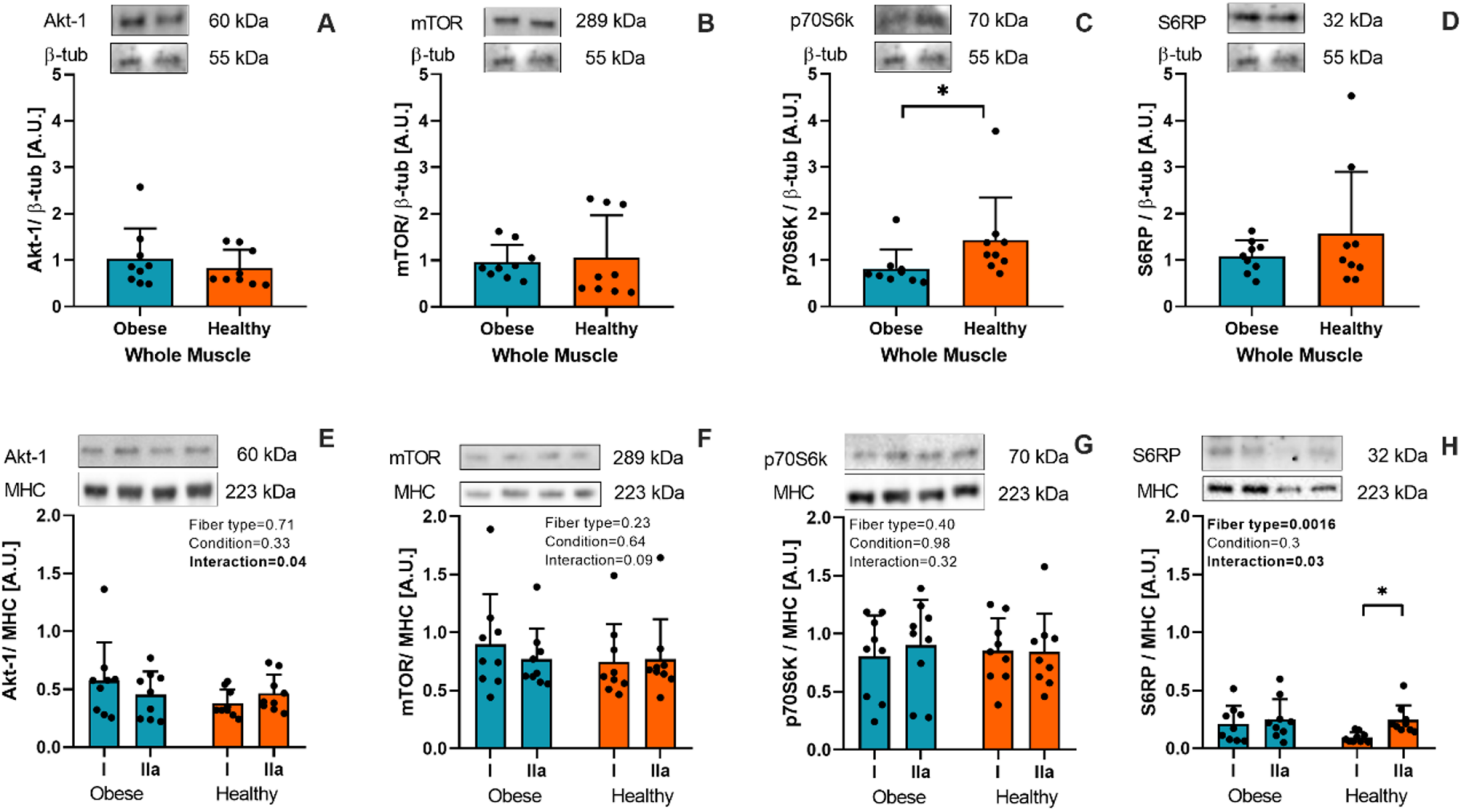
Total protein content of akt-1, mTOR, p70S6K, and S6RP of whole muscle homogenate (**A**-**D**) and in isolated type I and IIa muscle fibers (**E**-**H**) from obese (green) and healthy (orange) individuals. The membranes with whole muscle homogenate samples were cut into three sections at 100 kDa for mTOR; at 50–100 kDa range for p70S6K and β-tubulin; and under 50 kDa, for S6RP proteins. Because Akt-1 and β-tubulin have similar molecular weights, two gels were prepared in duplicate and run simultaneously to use β-tubulin as a loading control. For the membranes loaded with specific fiber samples, they were also cut into three sections at 100 kDa, for mTOR and MHC; at 50–100 kDa range, for Akt-1 and p70S6K; and under 50 kDa, for S6RP proteins. * : *P* < 0.05.

Healthy individuals showed 73% greater p70s6K protein levels than obese individuals in whole muscle homogenates (*P* = 0.008; Fig. 2C). However, no significant differences were found in other proteins (Akt-1, mTOR, and S6RP) evaluated in whole muscle homogenates. The S6RP protein exhibited higher levels in type IIa fibers compared to type I fibers (*P* < 0.05), which also demonstrated a significant interaction and fiber type effect (Fig. 2H).

### Total protein ubiquitination

**Fig. 3:**
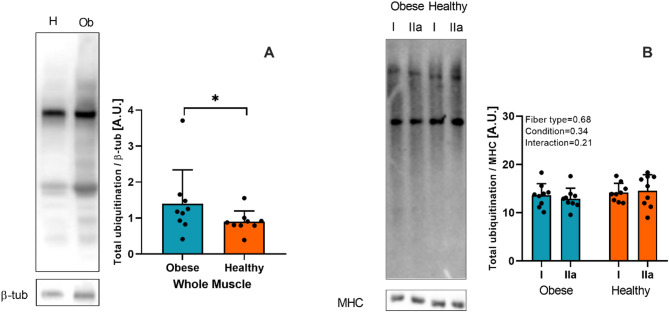
Total ubiquitination of whole muscle homogenate (**A**), and in isolated type I and IIa muscle fibers (**B**) from obese (green) and healthy (orange) individuals. For whole muscle two gels were loaded in duplicate and ran simultaneously. One of the gels was used to assess ubiquitination, while the duplicate gel was used to obtain a loading control. * : *P* < 0.05.

A 32% greater level of protein ubiquitination was found in the whole muscle of obese than in healthy individuals (*P* = 0.03), while no differences were found between fiber types (Fig. 3A and B).

### MAP kinases

**Fig. 4:**
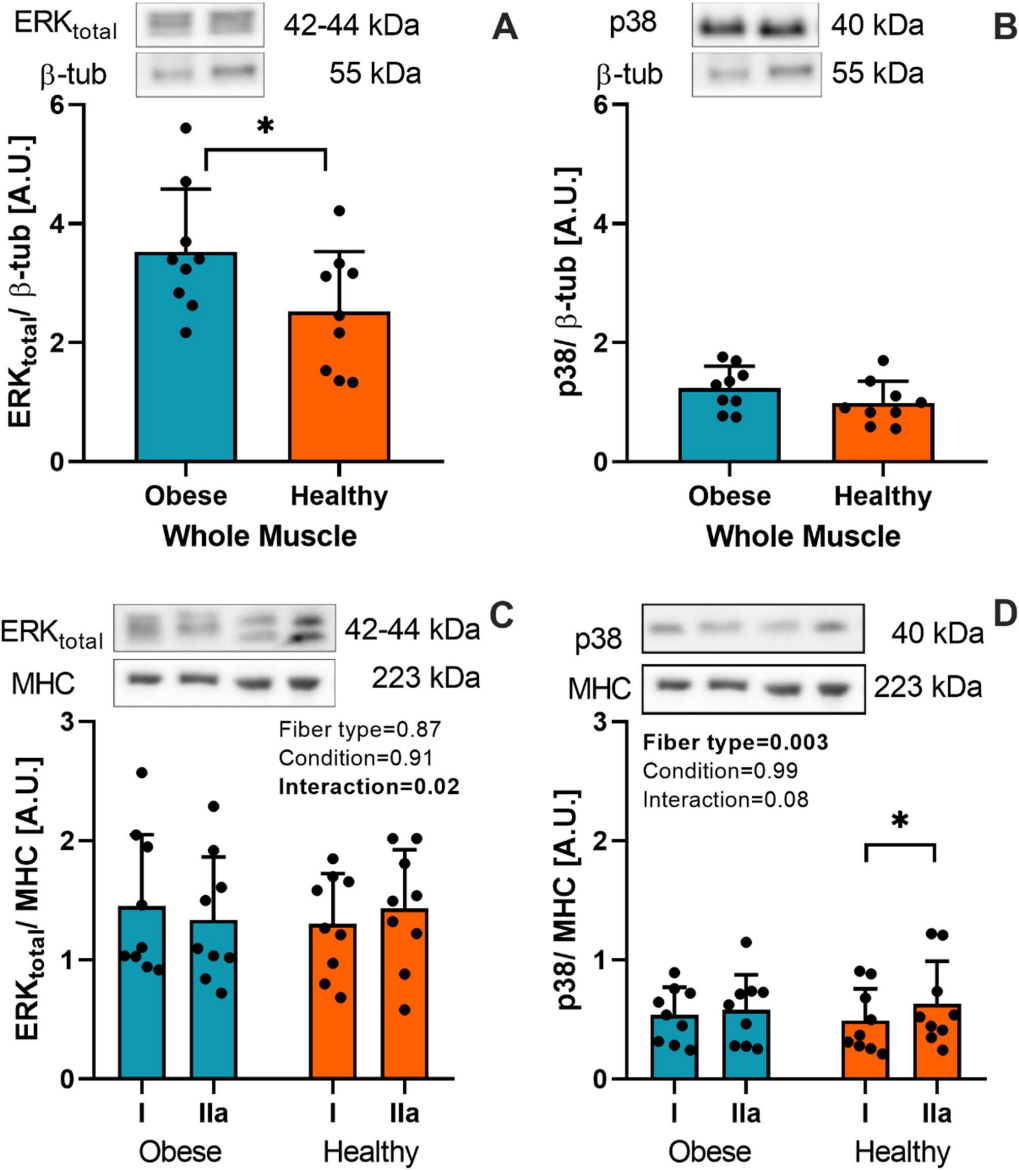
Total protein content of MAP-kinase ERK and p38 in whole muscle homogenate (**A**,** B**) and isolated type I and IIa muscle fibers (**C**,** D**). The membranes containing whole muscle homogenate samples were cut at the 50 kDa to assess β-tubulin and ERK. The membranes loaded with specific fiber samples were also cut at the 50 kDa mark to assess total ERK and at the 100 kDa mark to assess MHC. In both cases, the sections used to assess ERK antibodies were removed using corresponding buffers and then used to analyze p38 levels. * : *P* < 0.05.

A 29% greater total ERK1/2 was found in obese than in healthy individuals in whole muscle homogenates (*P* = 0.05; Fig. 4A). However, ERK1/2 expression was similar between fiber types (Fig. 4C). The total level of p38 in whole muscle homogenates was similar between obese and healthy individuals (Fig. 4B). However, 24% greater p38 levels were found in type IIa than in type I fiber in healthy individuals, evidencing a significant main effect of fiber type (*P* = 0.004; Fig. 4D).

### Mitochondrial complexes

**Fig. 5:**
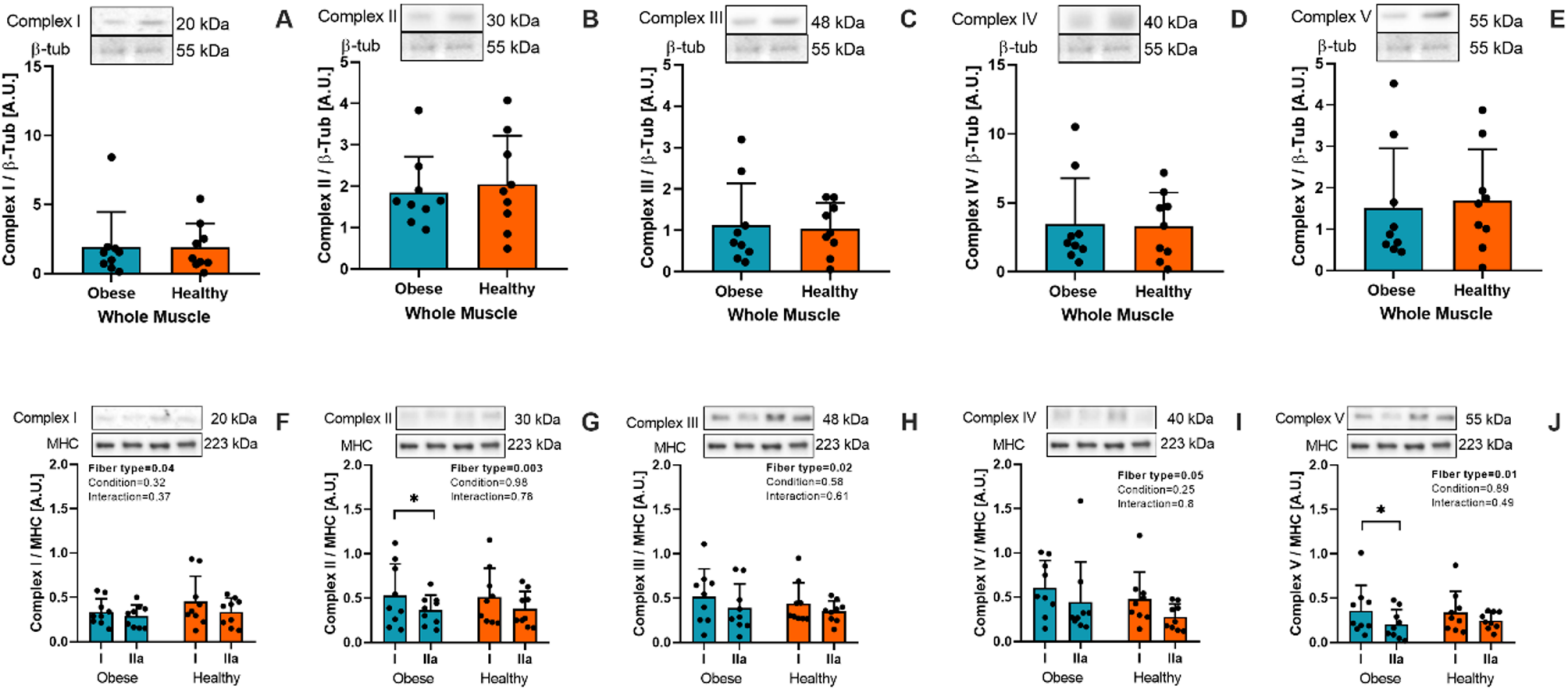
Total protein content of mitochondrial complexes I-V from whole muscle homogenate (**A**-**E**) and isolated type I and IIa muscle fibers (**F**-**J**) of obese (green) and healthy (orange) individuals. Two gels were loaded in duplicate for whole muscle OXPHOS evaluation. One of the gels was used to assess OXPHOS, while the duplicate gel was used to obtain β-tubulin. * : *P* < 0.05.

No differences were found in any OXPHOS subunits in muscle homogenate between obese and healthy individuals (Fig. 5A-E). As shown in Fig. 5G and J, greater levels of complex II and V (30.5% and 42.7%, respectively) were found in type I compared to type IIa fibers of obese individuals (*P* = 0.03 and *P* = 0.04, respectively). However, all subunits showed a significant main effect of fiber type, depicting greater protein content in type I muscle fibers.

### Other cell population

**Fig. 6:**
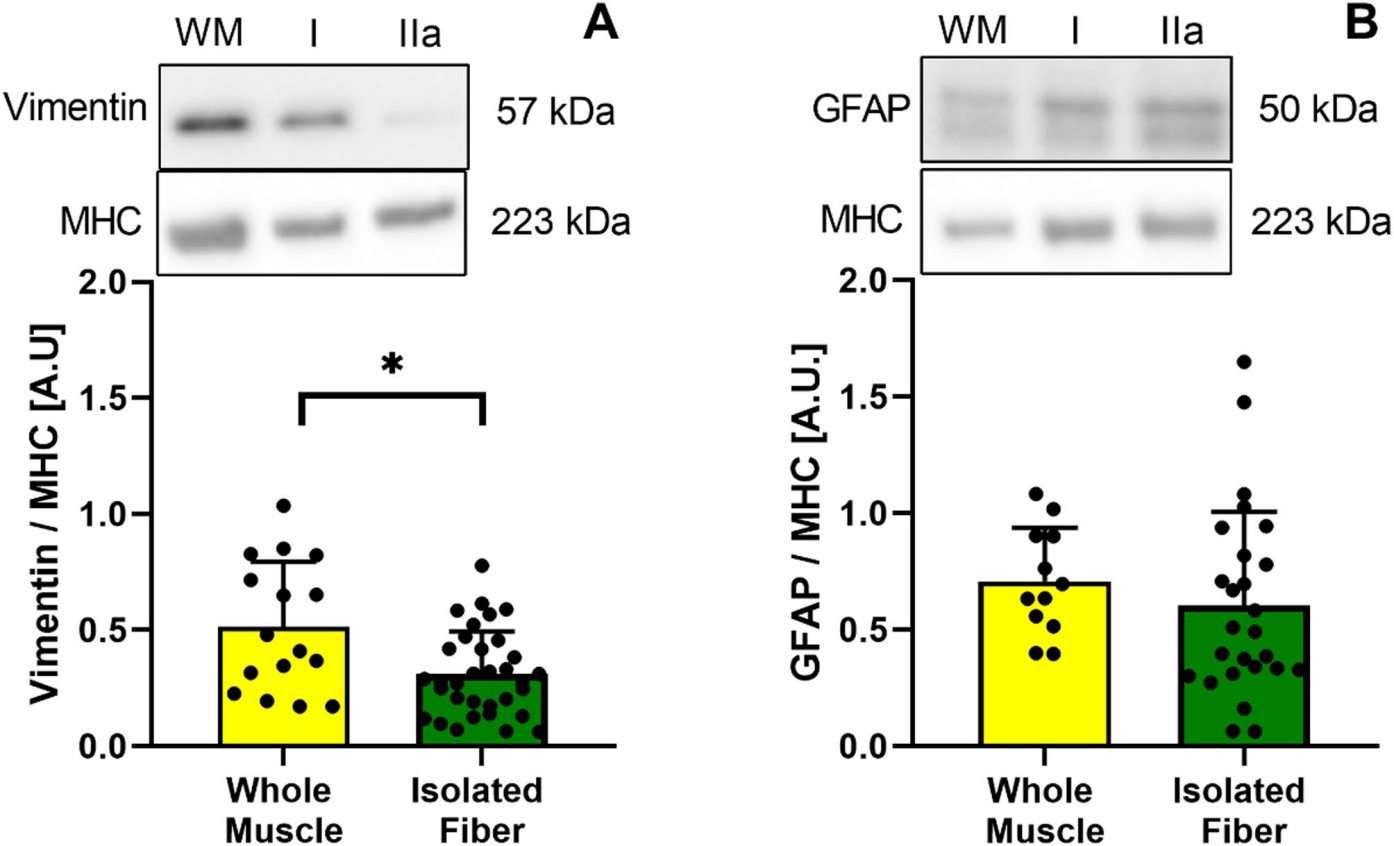
Total protein content of vimentin (**A**) and glial fibrillar acidic protein (GFAP) (**B**) from whole muscle samples and isolated muscle fibers normalized by MHC. * : *P* < 0.05.

GFAP levels were similar between whole muscle and isolated fibers. However, vimentin protein content was 38% lesser in isolated fibers than in whole muscle homogenates (*P* = 0.05; Fig. 6A).

### Correlations

**Fig. 7:**
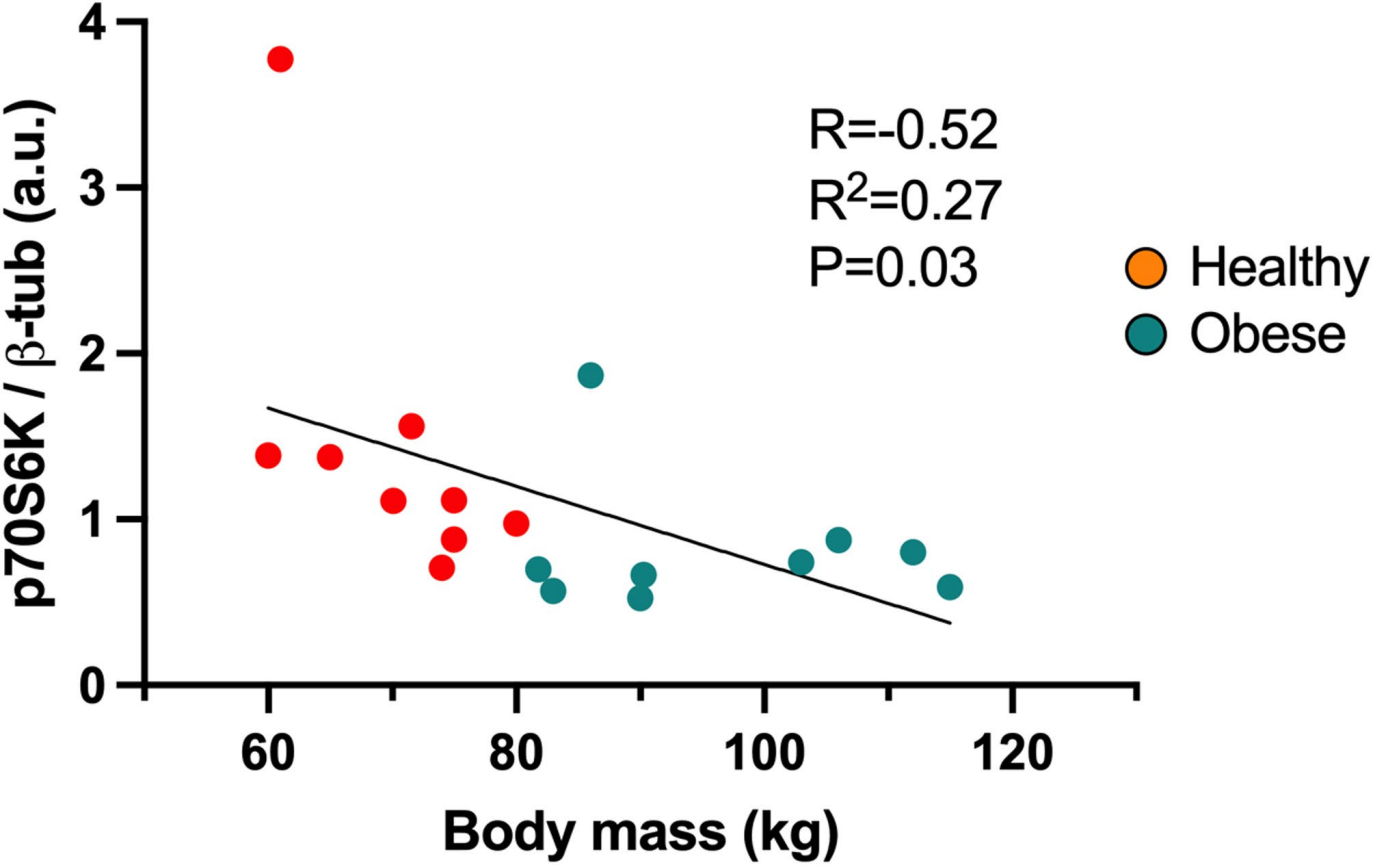
Correlation between body mass and whole muscle p70S6K protein levels in obese (green) and healthy (orange) individuals.

We found a moderate correlation (*R*=-0.52, *P* = 0.03) between the body mass of all participants (obese and healthy) and total content of p70S6K protein in whole homogenized samples (Fig. 7).

## Discussion

Our main findings revealed nutritional status-dependent and fiber type-specific changes in the proportion, size, and protein content of key metabolic proteins related to the mTORC1 pathway and degradation system, as well as in MAPK and OXPHOS proteins. In individuals with obesity, we found fewer type I than type IIa muscle fibers. Additionally, the cross-sectional area (CSA) of type I fibers was larger than that of type IIa fibers in individuals with obesity. Furthermore, the CSA of type I fibers in individuals with obesity was greater than that of type I fibers in healthy individuals. We also found a moderate association between body mass and p70S6K protein content. Interestingly, the vimentin protein was greatly expressed in whole homogenized samples compared to isolated fibers, which may show the presence of other non-muscle cell populations in the samples. Therefore, our hypothesis is accepted partially.

Our results align with a substantial amount of evidence that shows that obesity is associated with a reduced percentage of type I fibers in muscle^38^. This suggests a shift in muscle fibers of the skeletal muscle of individuals with obesity from slow-type I to fast-type II fibers^18^. It has been speculated that a long-term diet high in fat and sugar results in a “slow-to-fast” fiber type distribution, as documented by a decrease in MHC-I gene expression in skeletal muscle^13^. The muscle fibers’ phenotypic changes (Fig. 1) could be explained by modifications in signaling pathways related to metabolism, protein synthesis, and degradation previously described in individuals with obesity^10,22^. These phenotypic changes in fiber types have been described as a continuum determined by motor neuron activity, metabolism, and ribosomal activity^39^. However, reports of fiber-specific adaptations are still scarce in the literature. Hence, more studies should focus on specific adaptations in type I and II fibers in skeletal muscle.

Although a decrease in muscle protein synthesis, known as anabolic resistance, has been described in individuals with obesity^40,41^, some discrepancies in the literature have been documented, likely due to a lack of standardized participant grouping and determination differences^21^. Several studies have shown that the rate of protein synthesis at the whole muscle level is lower in humans with obesity compared with lean controls^15^. In line with that, we found greater protein levels of p70S6K in whole muscle homogenates in healthy compared to obese individuals (Fig. 2C). Although type I muscle fibers have significantly more ribosomal and proteasome-associated proteins, supporting a higher protein turnover rate^13^, we did not find fiber specific differences in Akt-1, mTOR, and p70s6Kprotein content (Fig. 2). The differences between protein content observed in whole muscle and specific muscle fibers could be due to whole muscle also included hybrid muscle fibers, which were discarded for type I and IIa isolated fibers analyses. Hence, a direct comparison of changes observed in whole muscle should consider this difference in fiber population. Interestingly, Edman et al. found greater levels of S6RP and eEF2 proteins in type II fibers compared to type I^42^. In contrast, others have shown no differences in protein fractional synthesis rates between fiber types^43^ or differences in p70S6K or S6RP proteins at rest in healthy individuals^44^. However, the net of muscle protein turnover also depends on protein degradation^45^, which has been far less studied in relation to muscle mass loss in obesity. Our findings showed increased TPU levels in muscle samples from individuals with obesity (Fig. 3A), consistent with previous reports linking this to higher protein degradation and muscle atrophy in the context of obesity^45^. For instance, Bollinger et al. showed increased ubiquitin-proteasome and autophagic proteolytic flux in primary human skeletal muscle cells from obese women^10^. However, no fiber-type specific differences were observed in the present study (Fig. 3B). We speculate that elevated TPU levels in the whole muscle of individuals with obesity may result from ubiquitination of other tissues (e.g., adipose tissue, nerves, vessels, connective tissue) and cell types (e.g., immune, FAPS, endothelial, and glial cells)^46^, which could contribute to degradation induced by low-grade inflammation commonly found in individuals with obesity^47^. Interestingly, we found that the total protein content of p70S6K was negatively correlated with body mass (Fig. 7), which may shed light on the disrupted protein synthesis pathway in individuals with obesity. However, other degradation pathways and protein phosphorylation states are necessary to fully elucidate muscle mass loss; hence, further research is warranted.

MAPK proteins are implicated in protein synthesis pathways in skeletal muscle^48^; we found elevated levels of ERK protein in individuals with obesity (Fig. 4A). Elevated levels of total ERK have also been reported in obesity^49^, which may contribute to insulin resistance^50^. As no significant fiber-type differences were found here, we speculate that the increased ERK levels may originate from non-muscle cells, such as immune cell infiltration, adipose tissue^51^, and vascular endothelial cells^46^. As vimentin is virtually absent in normal human muscle^37^, greater levels of vimentin in whole muscle homogenates may represent larger heterogeneity in cell populations within samples (Fig. 6), which could interfere with molecular analyses performed on whole muscle homogenates. We also found that p38 was more abundant in type IIa fibers from healthy muscle samples (Fig. 4D). p38 MAPK is essential for regulating the critical processes that enable skeletal muscle to adapt to the metabolic demands and energy requirements during exercise^52^. On the other hand, it has been reported that oxidative stress is involved in the chronic activation of p38 MAPK in obesity, a process linked to insulin resistance in skeletal muscle, but its role is still controversial^53^.

Oxidative capacity is determined by mitochondrial function, which is at least in part determined by the mitochondrial complexes for energy production^54^. However, we did not find differences in mitochondrial complexes (i.e., OXPHOS) subunits protein content in whole muscle between individuals with obesity and healthy controls (Fig. 5A-E). Nevertheless, we found a significant main effect of fiber type when fiber-specific analyses were performed (Fig. 5F-J), which aligns with previous findings from proteomic^39,55,56^ and animal studies^57,58^. However, Samjoo et al. did not find differences in COX IV protein content between lean and obese individuals analyzed in whole muscle samples^59^. This may be due to variations in the degree of obesity or coexisting conditions, such as insulin resistance^57^. We found fiber-specific differences in mitochondrial complex II and V of individuals with obesity, with type I fibers showing higher levels than type IIa fibers (Fig. 5G). Although the main sites involved in mitochondrial ROS production are believed to be localized at complexes I and III, succinate-dependent ROS production by complex II has also been reported to contribute in skeletal muscle^60^, which has been likely linked to differential oxidative stress in obesity^61^. Interestingly, a previous study in healthy young participants showed lesser protein content of CI, CII, and CIV in type II muscle fibers, while CII protein content was greater in type II fibers^62^. Thus, this finding may reveal the importance of mitochondrial complex II and V in skeletal muscle fibers in obesity. These findings warrant further research.

This study was not free of limitations. As shown in Table 1, although BMI and body weight were greater in the obese group, we also found that this group was older and trended toward lower physical activity levels. As age and reduced physical activity have been shown to induce further impairments in skeletal muscle metabolism, these differences may also include some variability, and our results should be interpreted cautiously.

In conclusion, heterogeneity of muscle fiber types exists in individuals with obesity, differing from that of healthy individuals. This study provided emerging evidence that some modifications in the skeletal muscle of individuals with obesity occur in a fiber-specific manner. Our findings suggest that obesity impairs protein synthesis pathways while increasing degradation. This change may be linked to a fiber-specific loss of oxidative metabolism-related proteins in type IIa fibers.

## Electronic supplementary material

Below is the link to the electronic supplementary material.

Supplementary Material 1

Supplementary Material 2

Supplementary Material 3

## Data Availability

Data is provided within the manuscript or supplementary information files.
