# Supplementary material for: Fiber-specific differences in protein content of pathways related to mTORC1 signaling and oxidative metabolism in individuals with obesity
Source: Sci Rep. 2025 Jul 4;15:23839. doi: 10.1038/s41598-025-09169-7 (PMC12229446; doi:10.1038/s41598-025-09169-7)
Supplement: Supplementary file 1 — Supplementary Material 1 [file 41598_2025_9169_MOESM1_ESM.pdf]

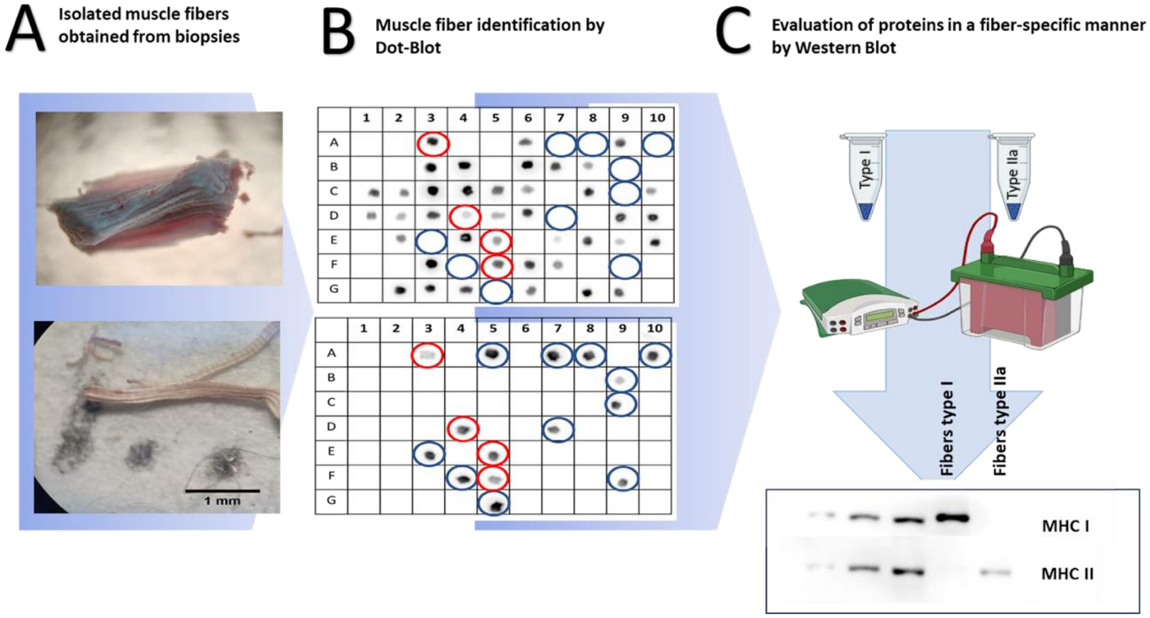

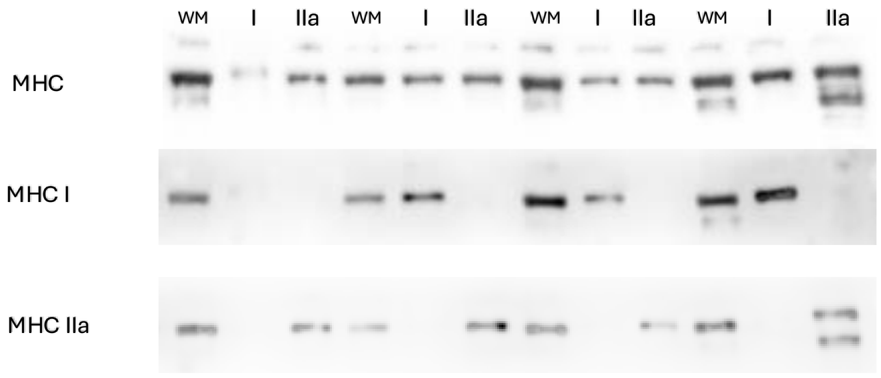

Supplementary Figure 1: The upper panel shows the workflow performed in muscle fiber isolation. (A) freeze-dried muscle was used for mechanical isolation of single fibers using forceps under the microscope. (B) Fiber type identification was performed by dot blot; fibers positive for both (or none) myosin heavy chain I and IIa were discarded from the study. (C) Specific fibers were polled and used for Western blot analysis.

The lower panel shows confirmation of muscle fiber typing by western blot in which total myosin heavy chain (MHC), type I (MHC I), and type IIa (MHC IIa) were marked in whole muscle homogenate (WM) and their respective pool of type I and IIa fibers.
