# Supplementary material for: Fiber-specific differences in protein content of pathways related to mTORC1 signaling and oxidative metabolism in individuals with obesity
Source: Sci Rep. 2025 Jul 4;15:23839. doi: 10.1038/s41598-025-09169-7 (PMC12229446; doi:10.1038/s41598-025-09169-7)
Supplement: Supplementary file 2 — Supplementary Material 2 [file 41598_2025_9169_MOESM2_ESM.pdf]

AKT-1/mTOR/p70S6k/S6RP Whole Muscle

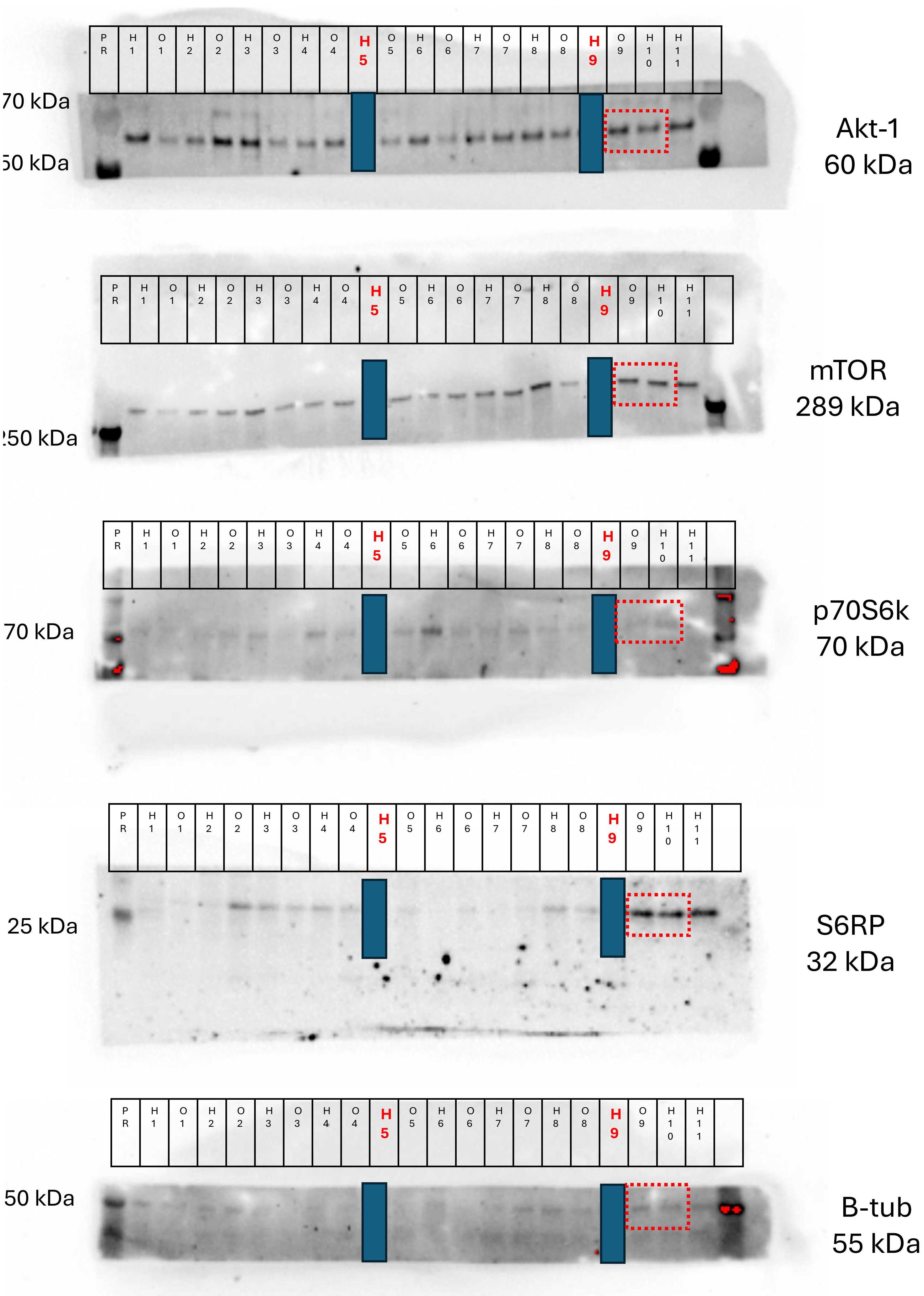

mTOR isolated fibers

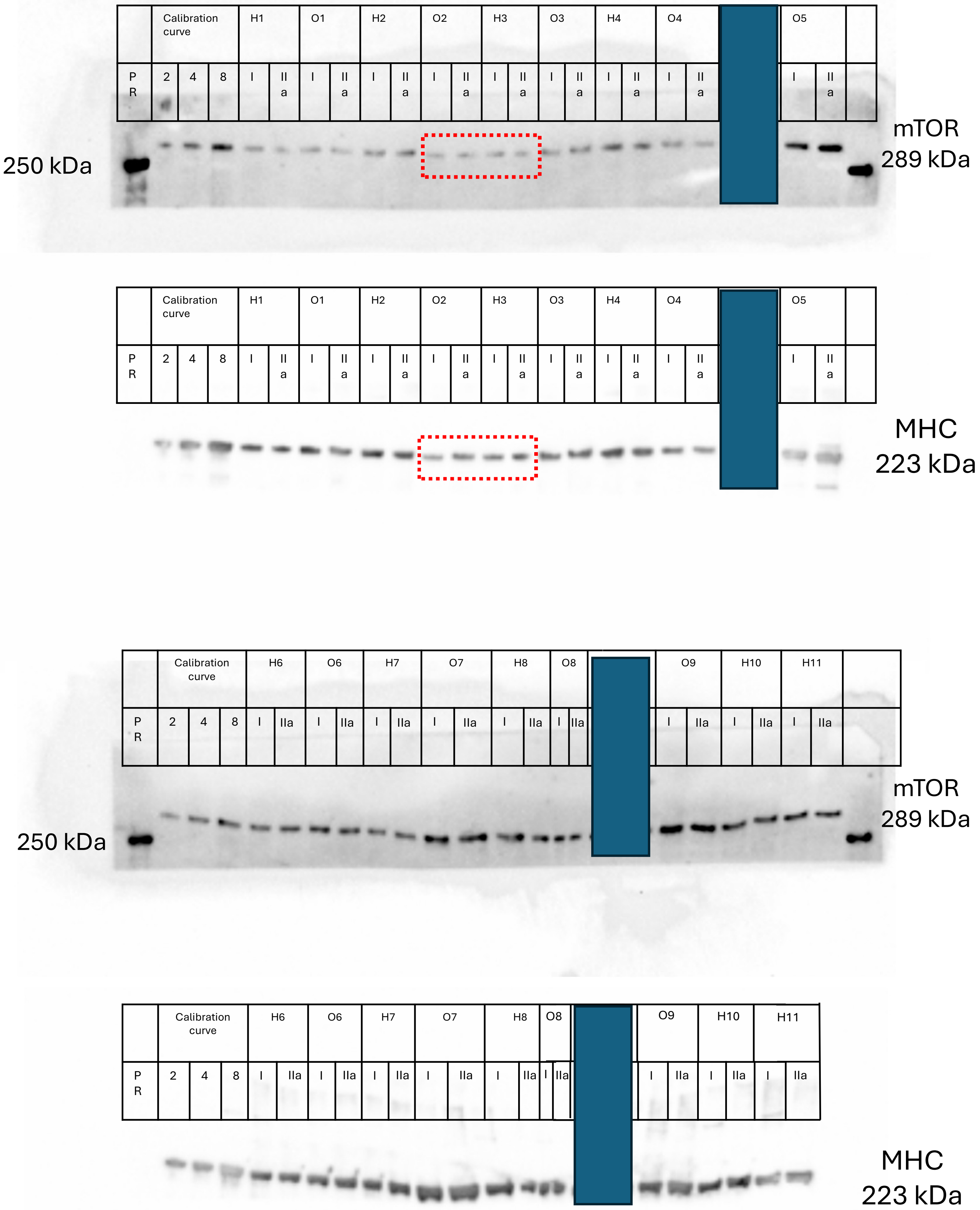

AKT-1 isolated fibers

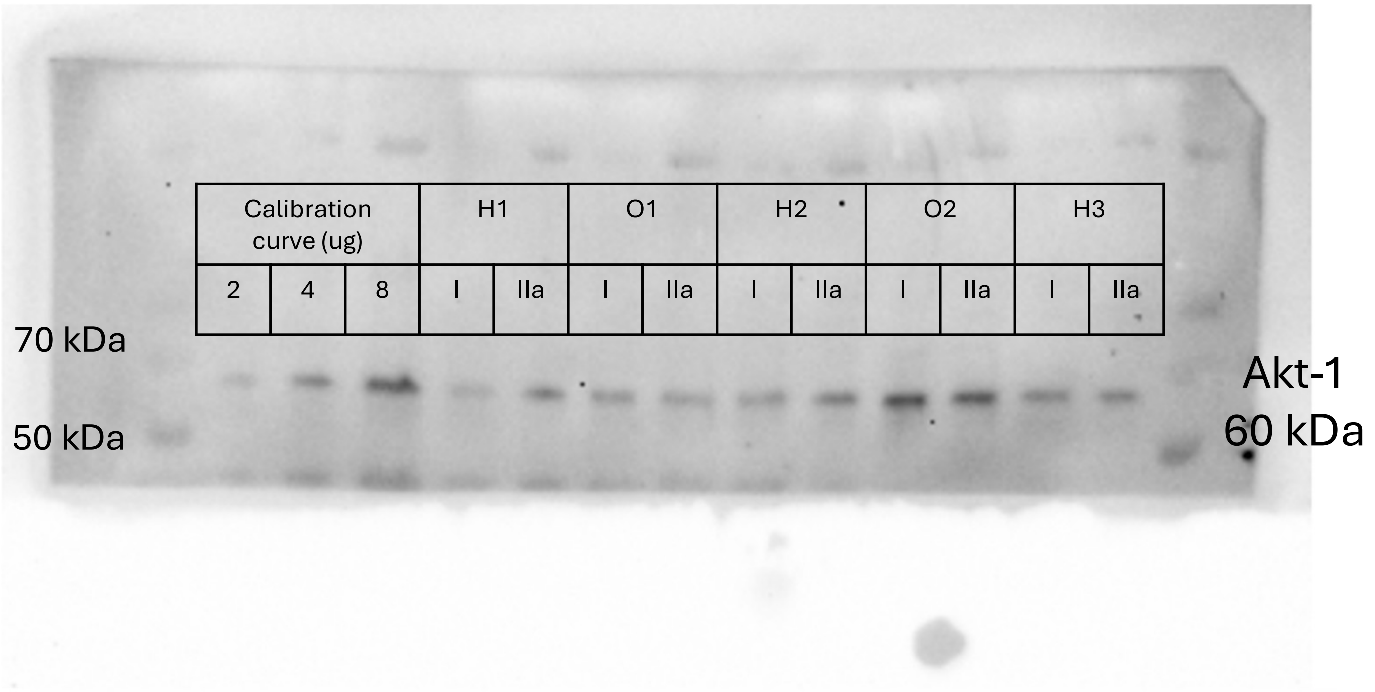

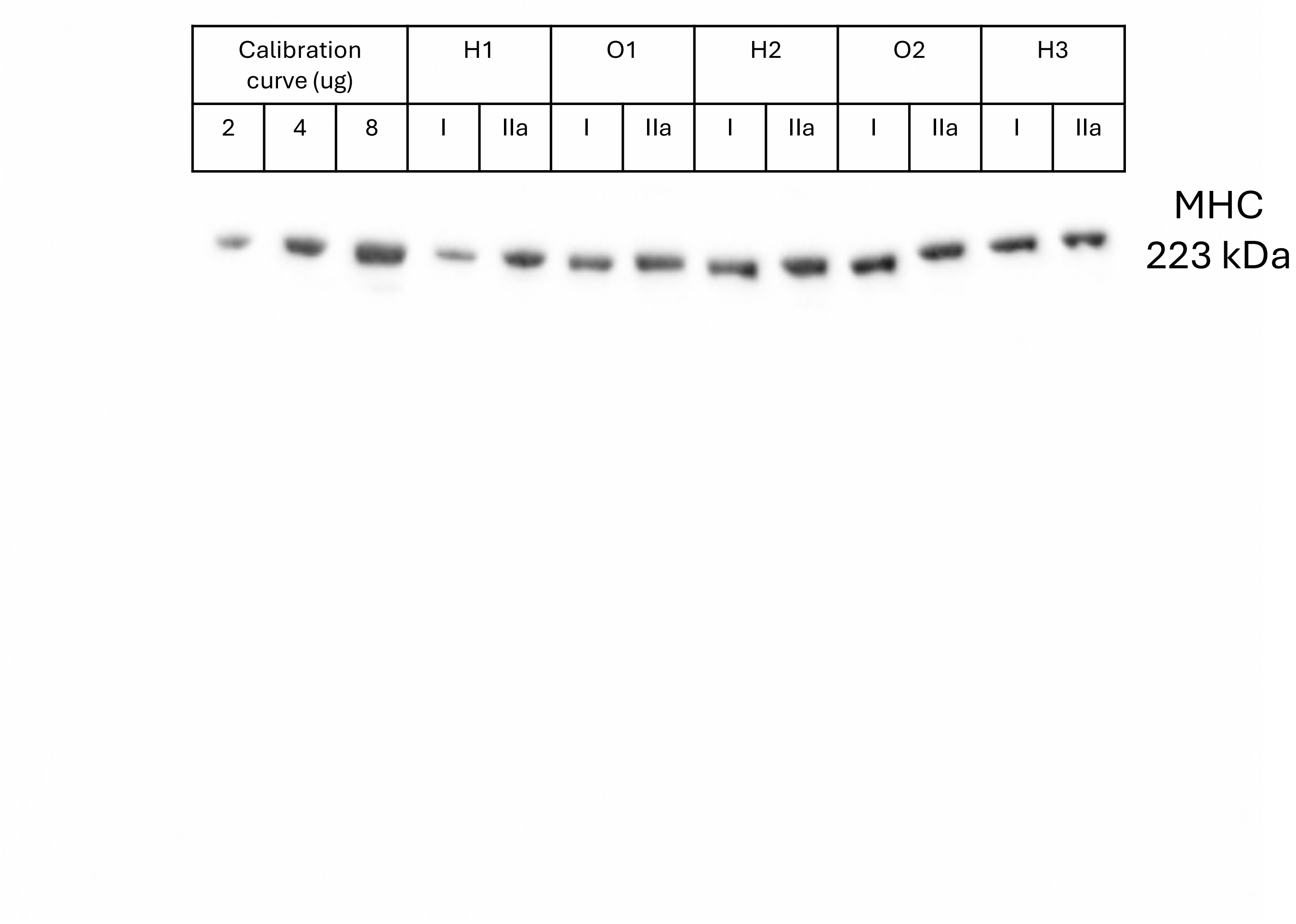

AKT-1 isolated fibers

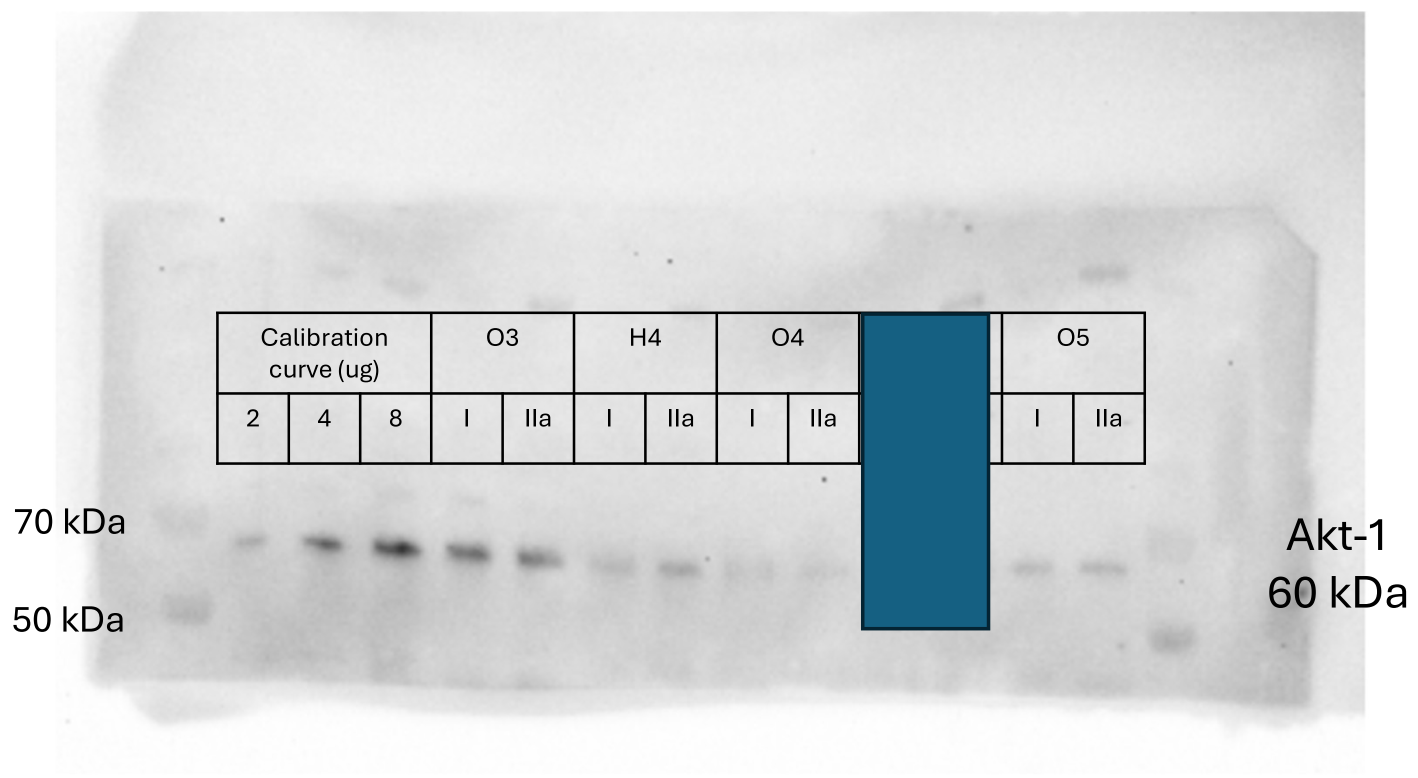

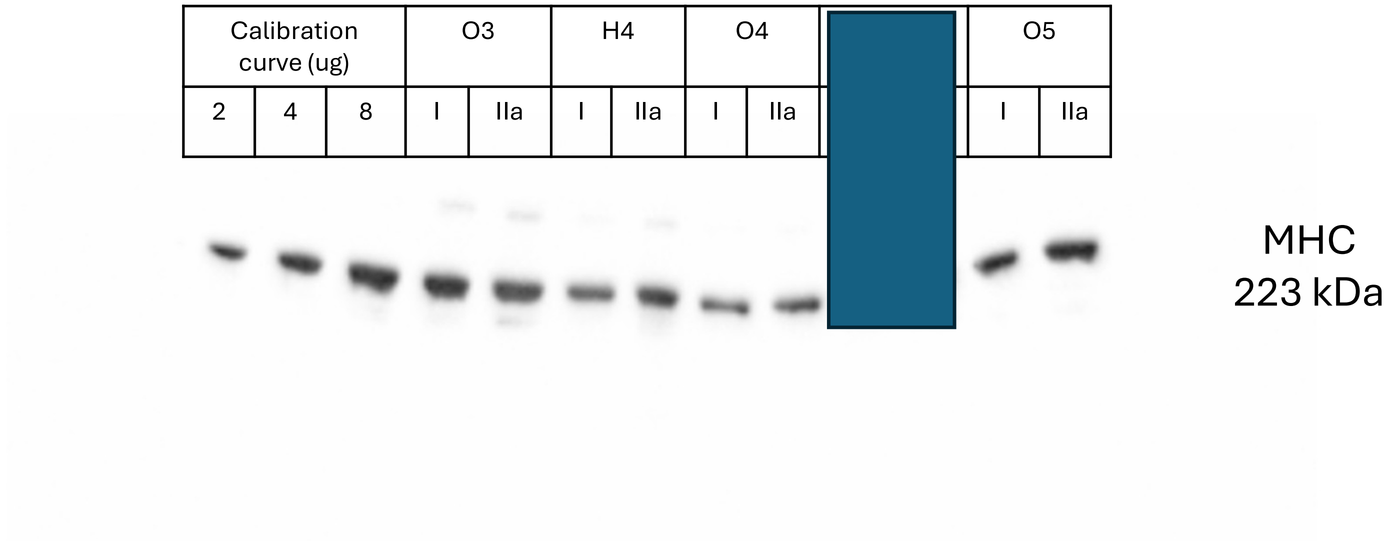

AKT-1 isolated fibers

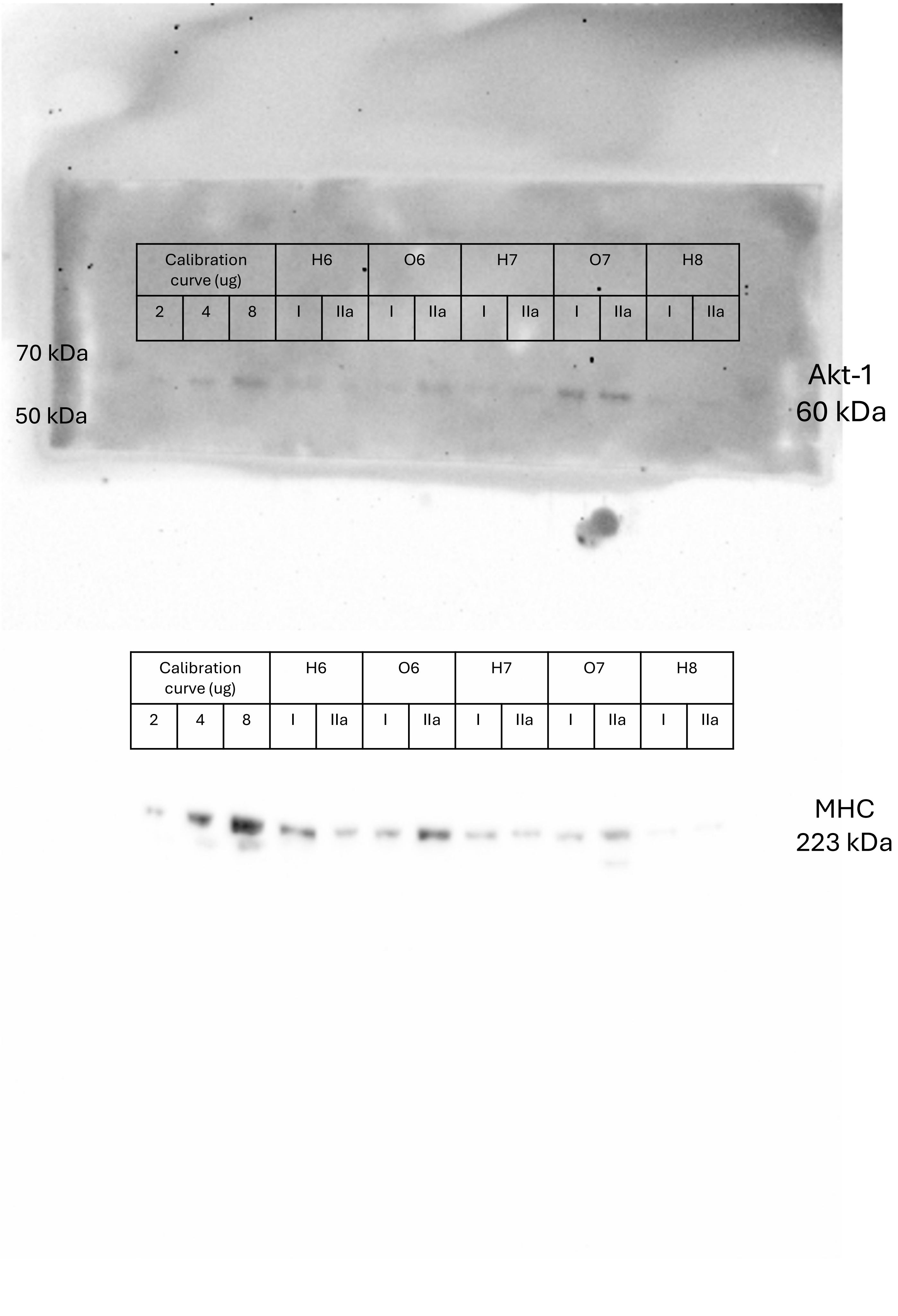

AKT-1 isolated fibers

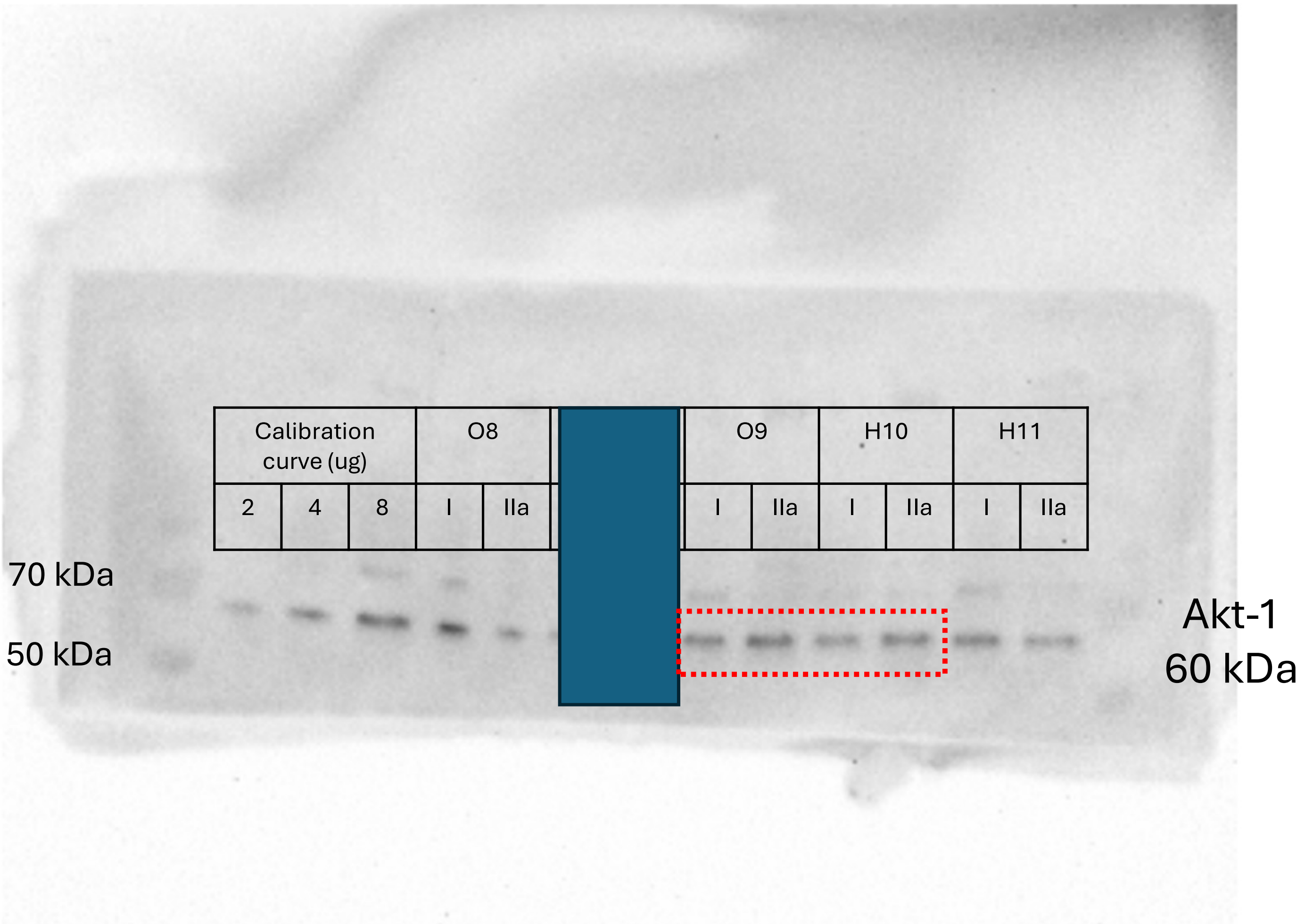

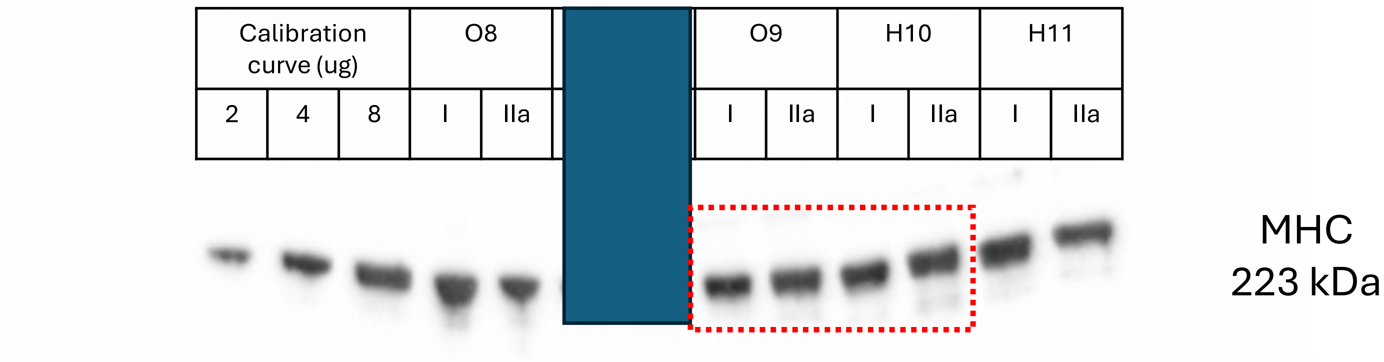

P70S6K isolated fibers

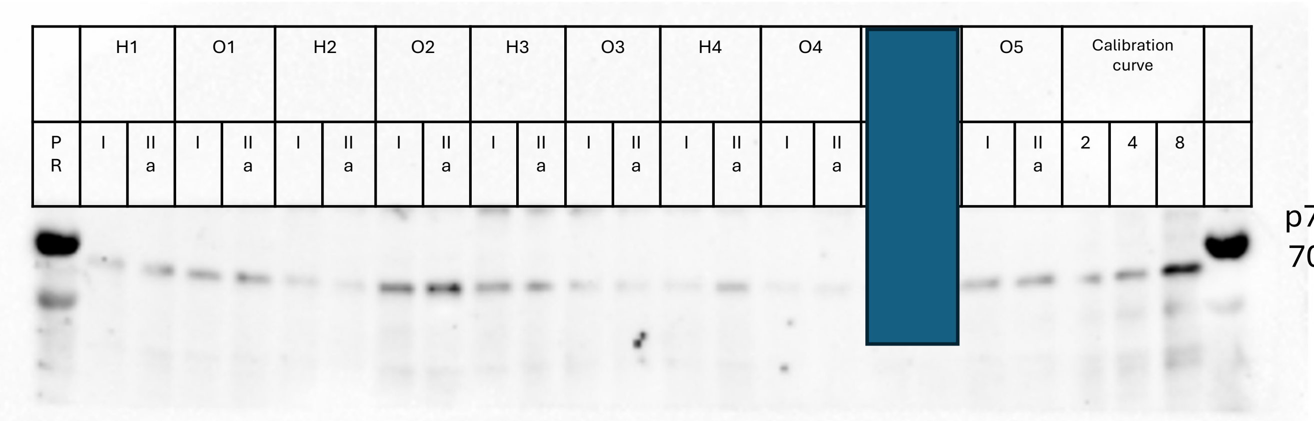

p70S6k
70 kDa

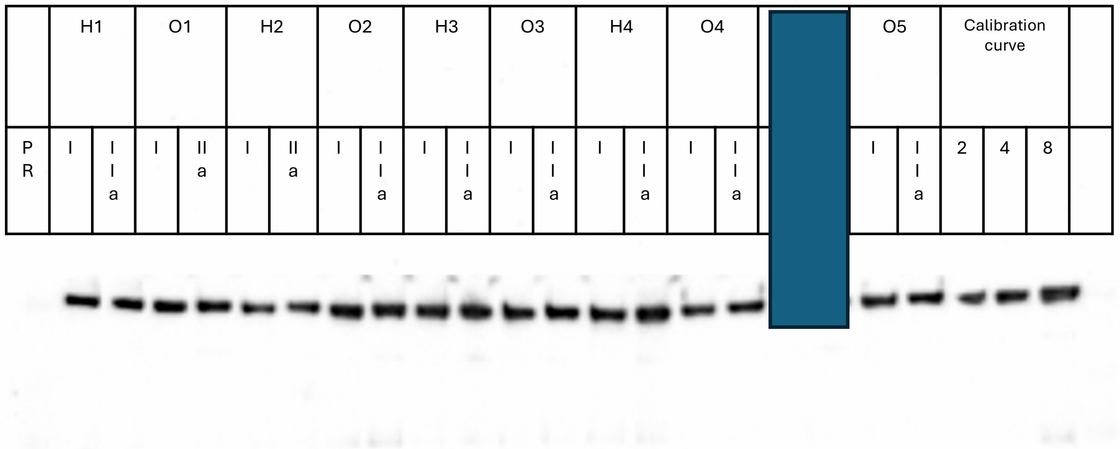

MHC
223 kDa

P70S6K isolated fibers

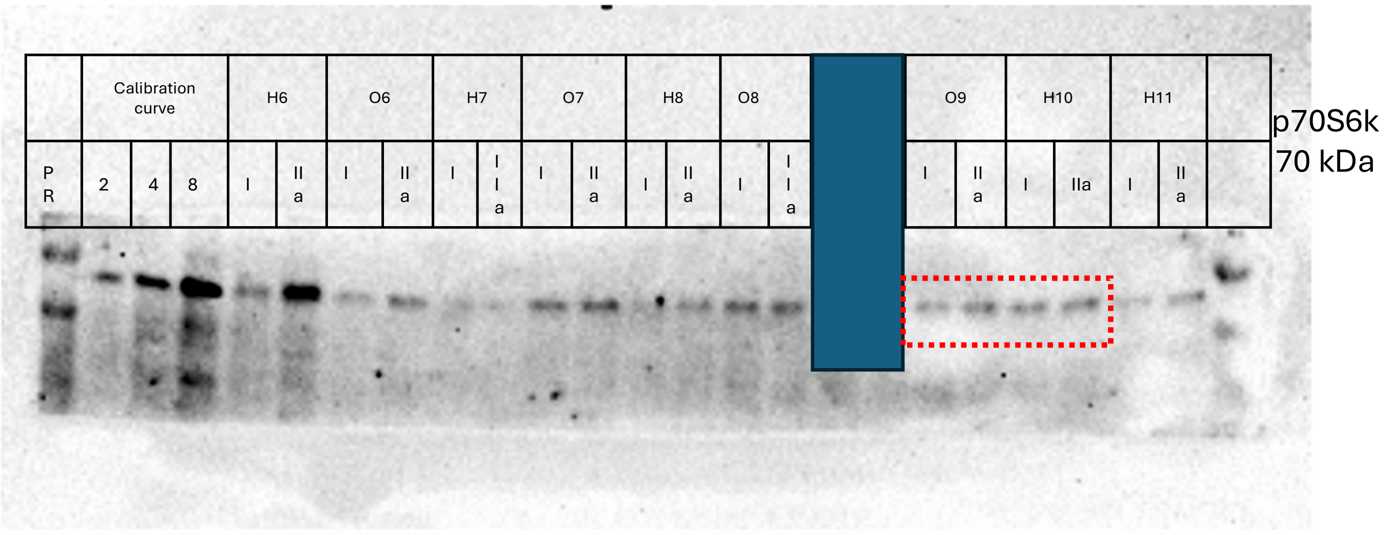

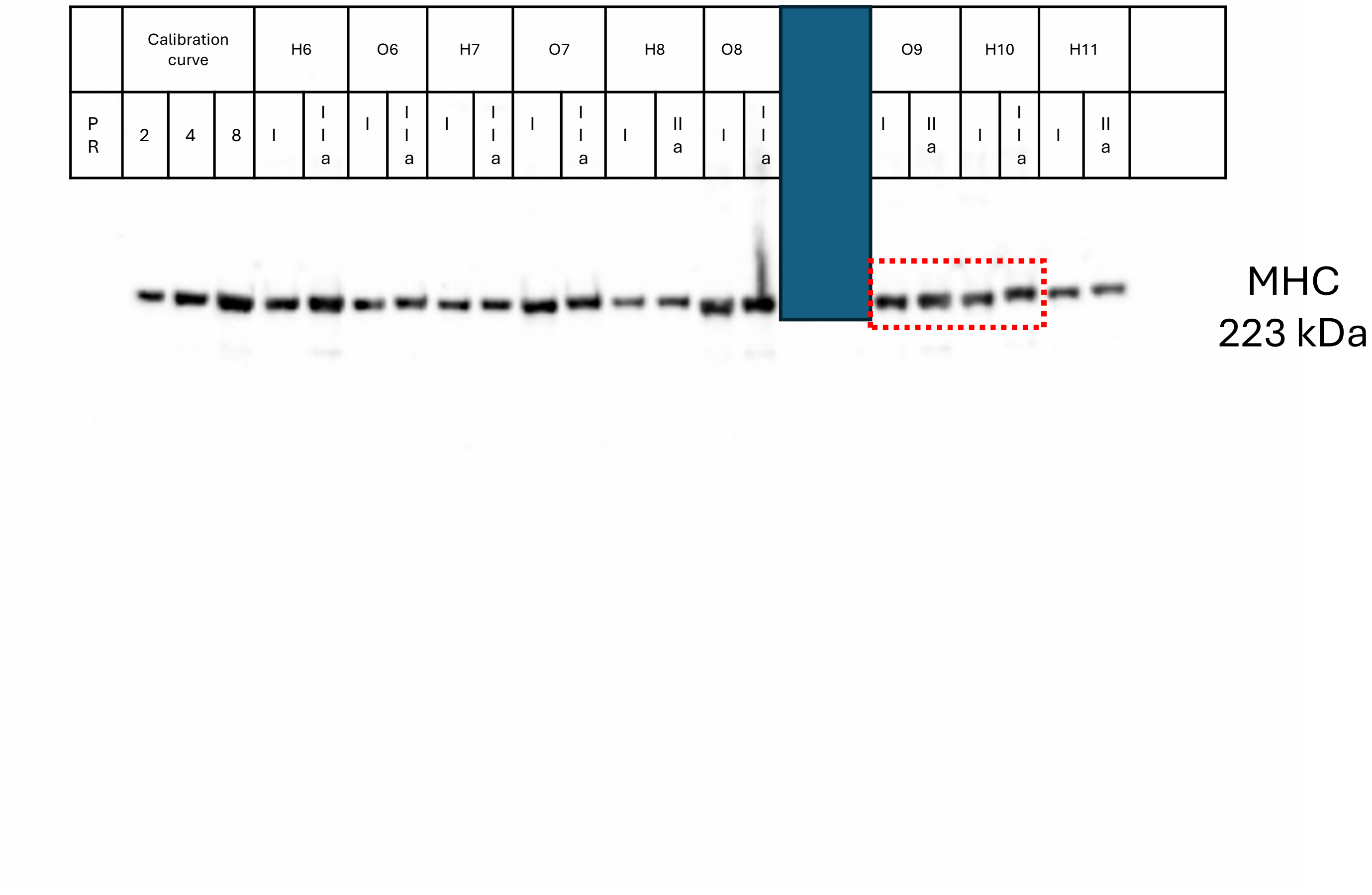

S6RP isolated fibers

Calibration curve (ug)			H1		O1		H2		O2		H3	
2	4	8	I	IIa	I	IIa	I	II a	I	IIa	I	IIa

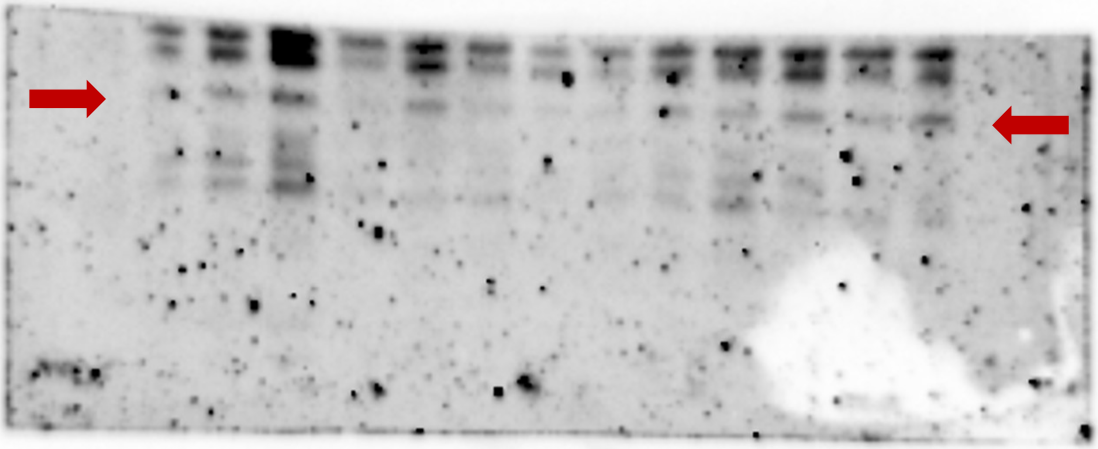

S6RP
32 kDa

Calibration curve (ug)			H1		O1		H2		O2		H3	
2	4	8	I	IIa	I	IIa	I	II a	I	IIa	I	IIa

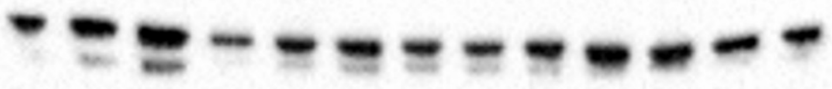

MHC
223 kDa

S6RP isolated fibers

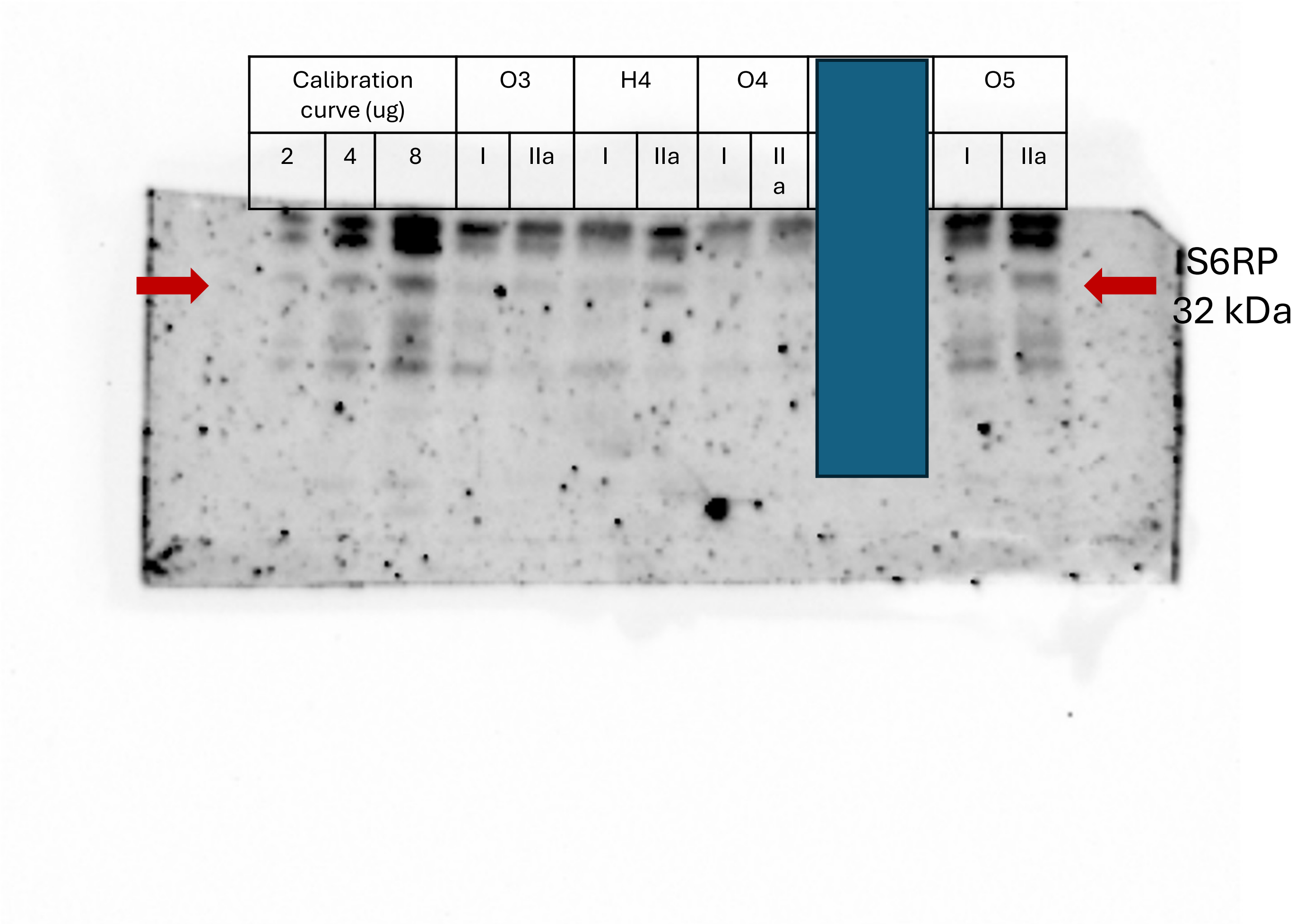

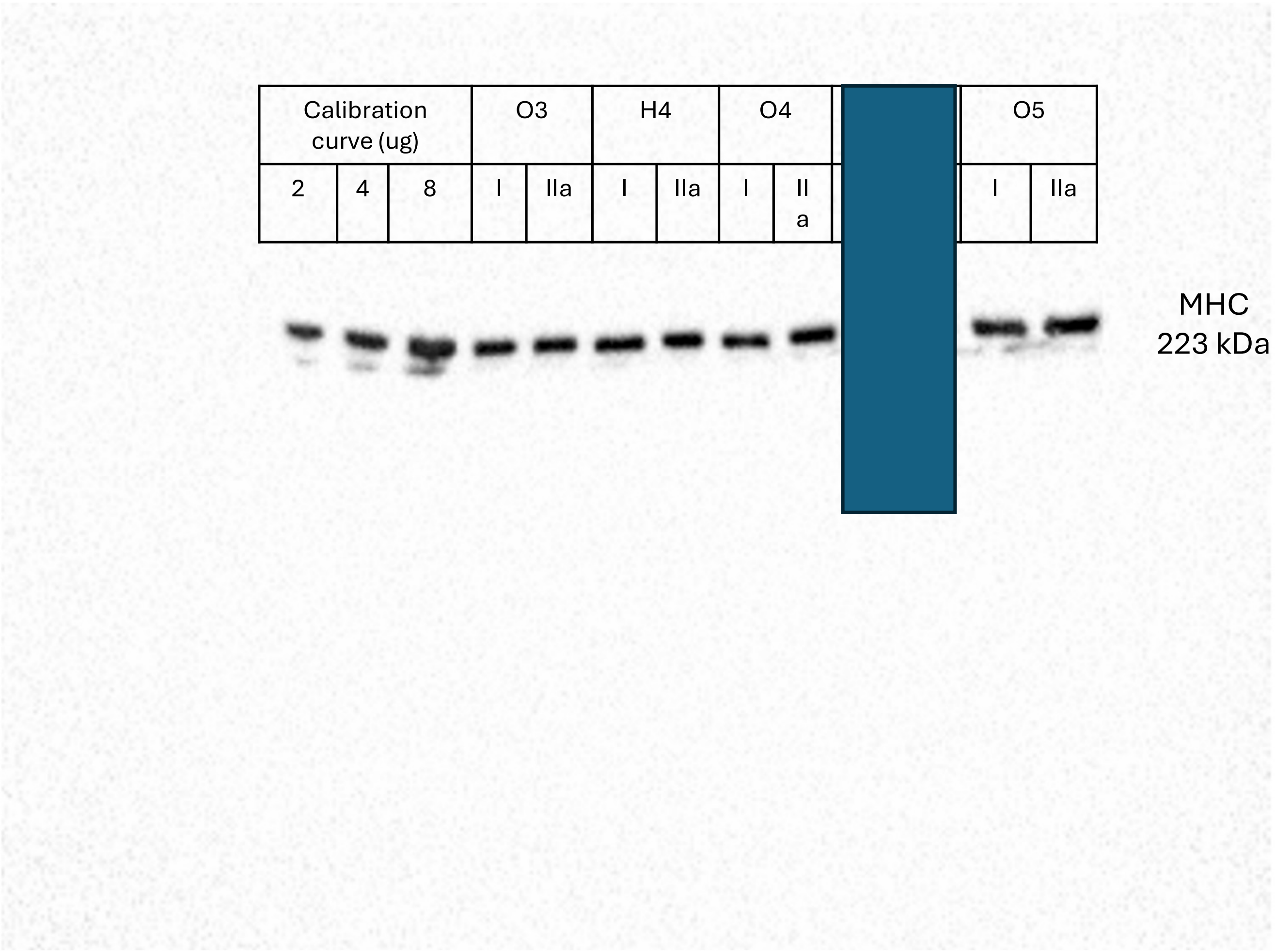

S6RP isolated fibers

Calibration curve (ug)			H6		O6		H7		O7		H8	
2	4	8	I	IIa	I	IIa	I	II a	I	IIa	I	IIa

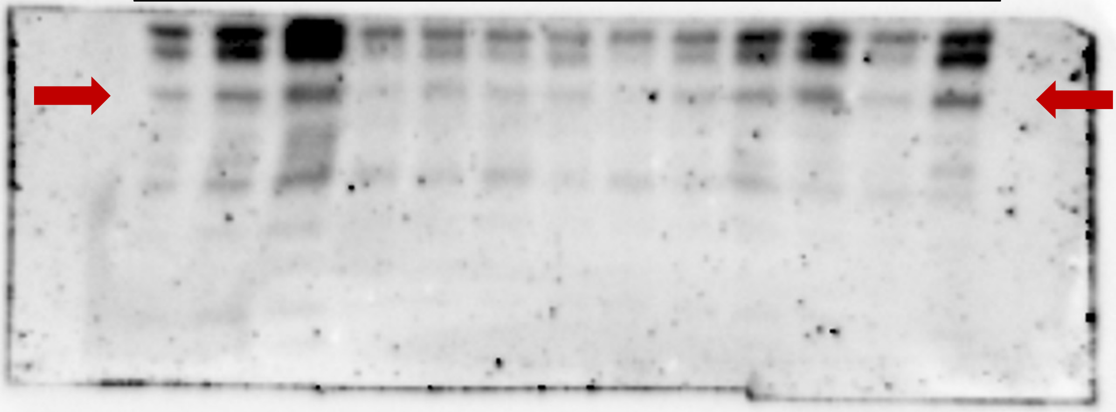

Calibration curve (ug)			H6		O6		H7		O7		H8	
2	4	8	I	IIa	I	IIa	I	II a	I	IIa	I	IIa

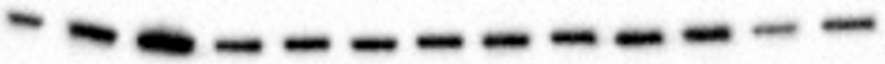

S6RP isolated fibers

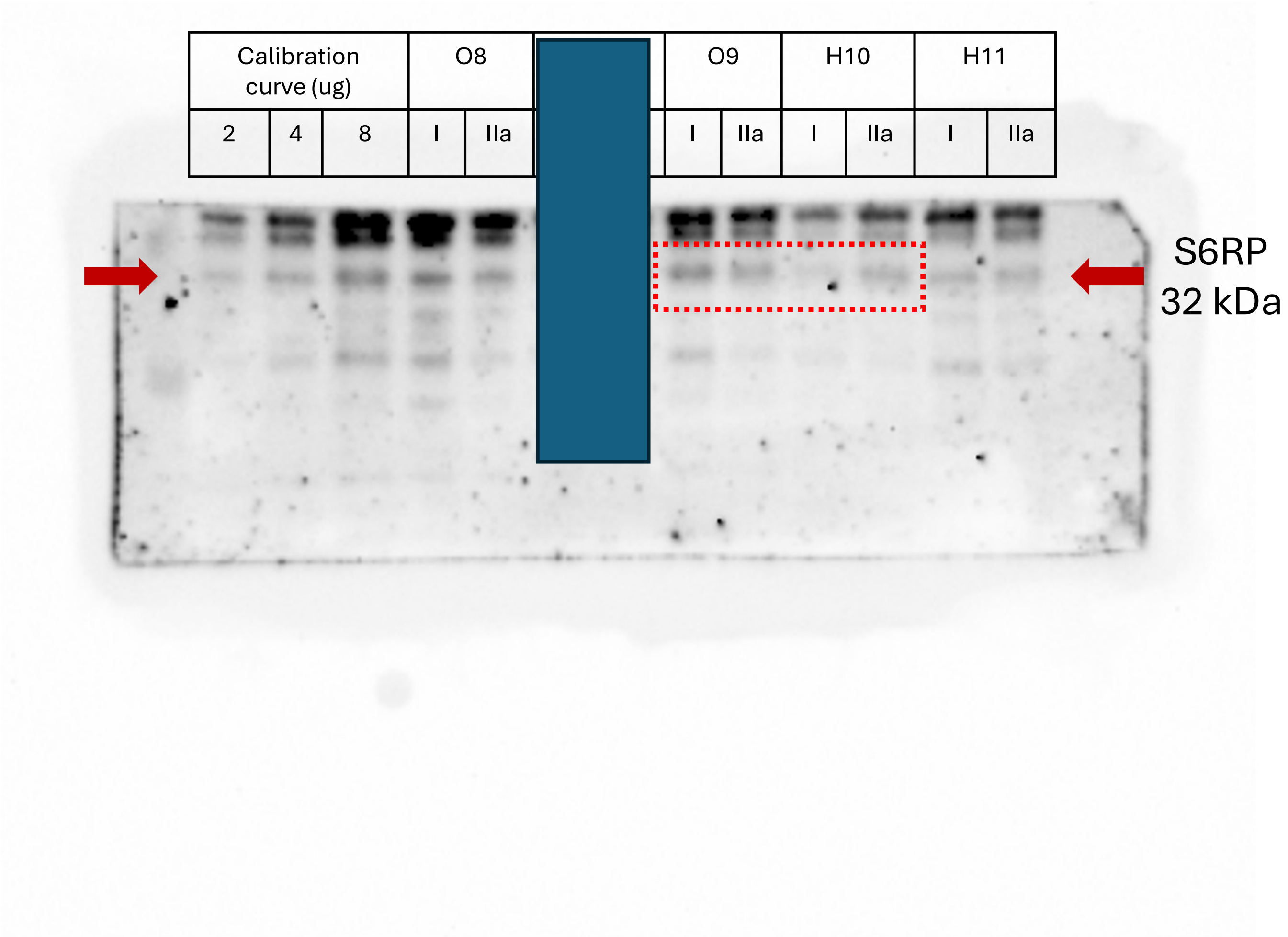

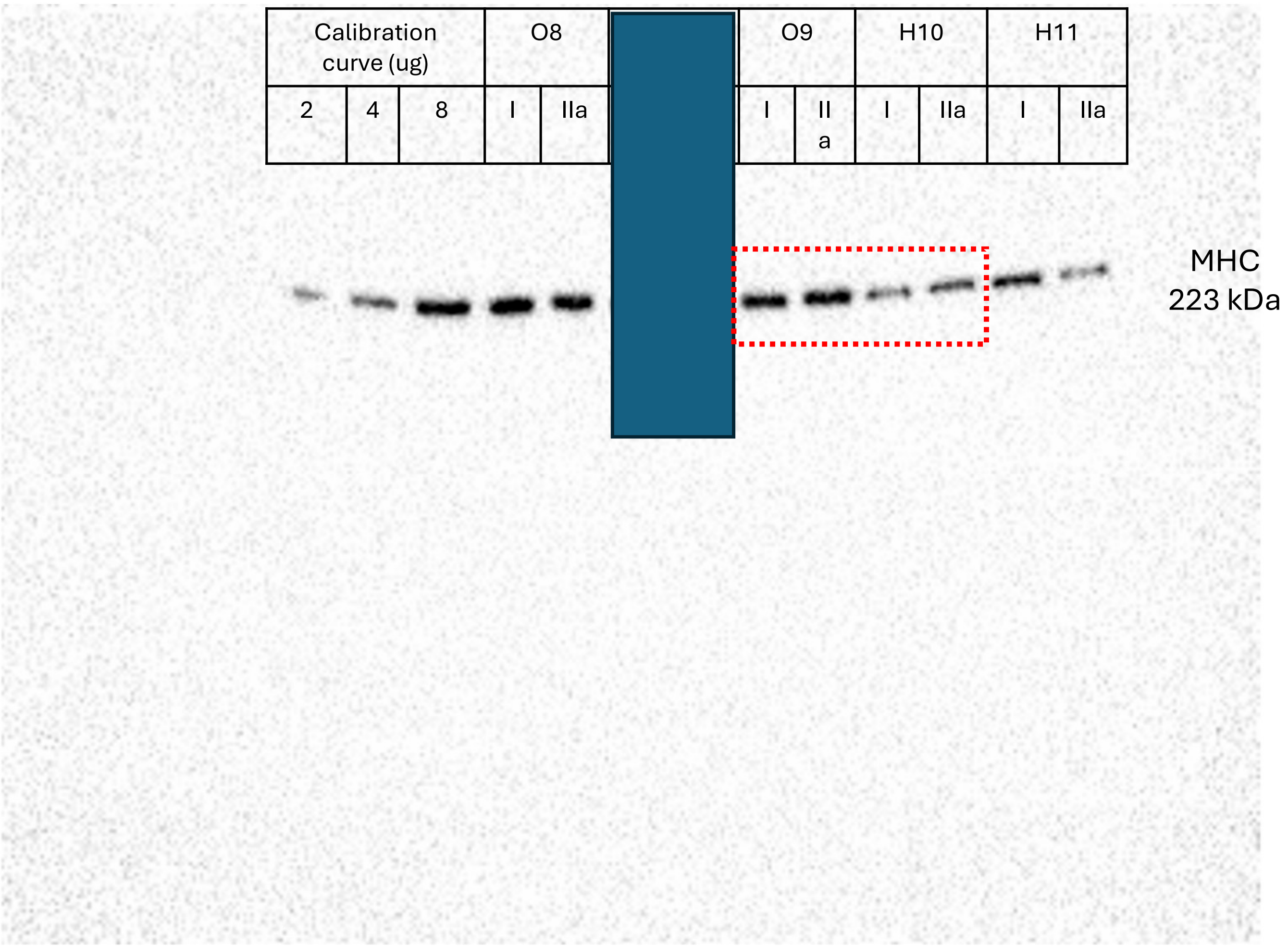

Ubiquitination Isolated fiber

	Calibration curve			H1		O1		H2		O2		H3		O3		H4		O4		[redacted]	O5		
PR	2	4	8	I	IIa	I	IIa	I	IIa	I	IIa	I	IIa	I	IIa	I	IIa	I	IIa	[redacted]	I	IIa	

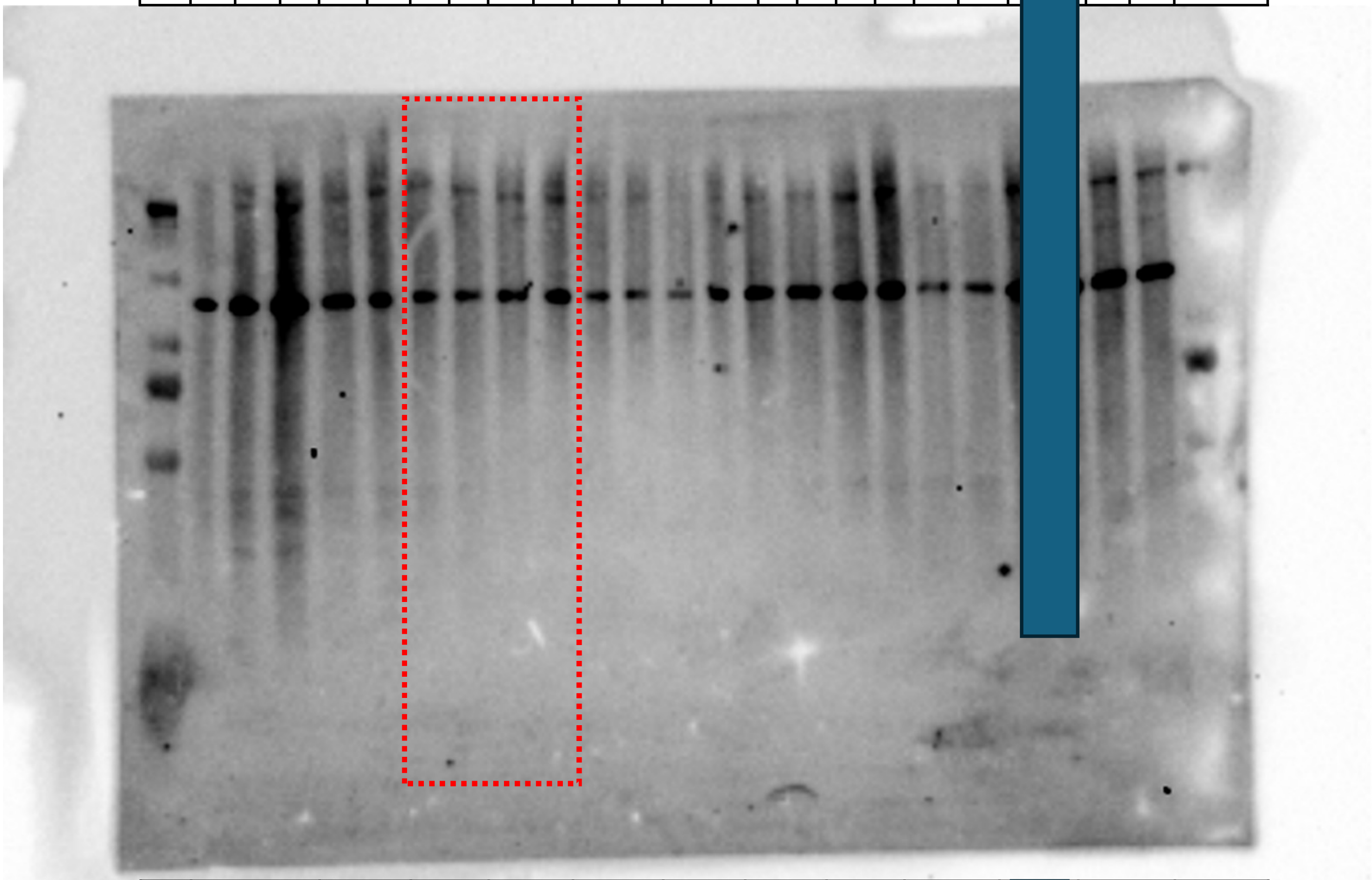

	Calibration curve			H1		O1		H2		O2		H3		O3		H4		O4		[redacted]	O5		
PR	2	4	8	I	IIa	I	IIa	I	IIa	I	IIa	I	IIa	I	IIa	I	IIa	I	IIa	[redacted]	I	IIa	

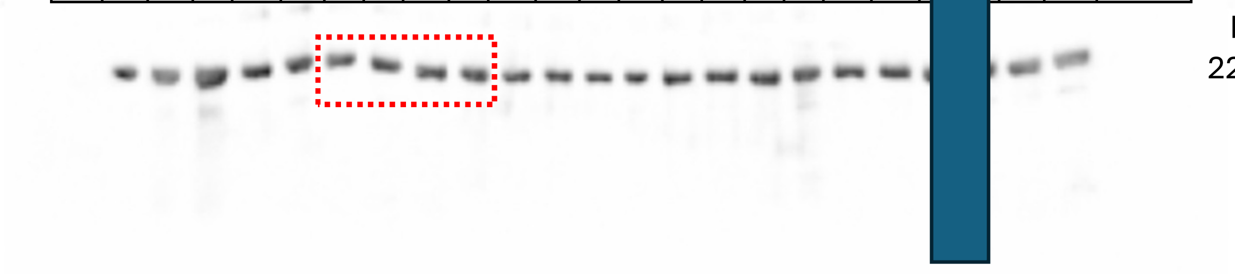

MHC
223 kDa

Ubiquitination Isolated fiber

	Calibration curve			H6		O6		H7		O7		H8		O8		[redacted]	O9		H10		H11		
P R	2	4	8	I	I I a	I	I I a	I	I I a	I	I I a	I	II a	I	I I a	[redacted]	I	II a	I	I I a	I	II a	

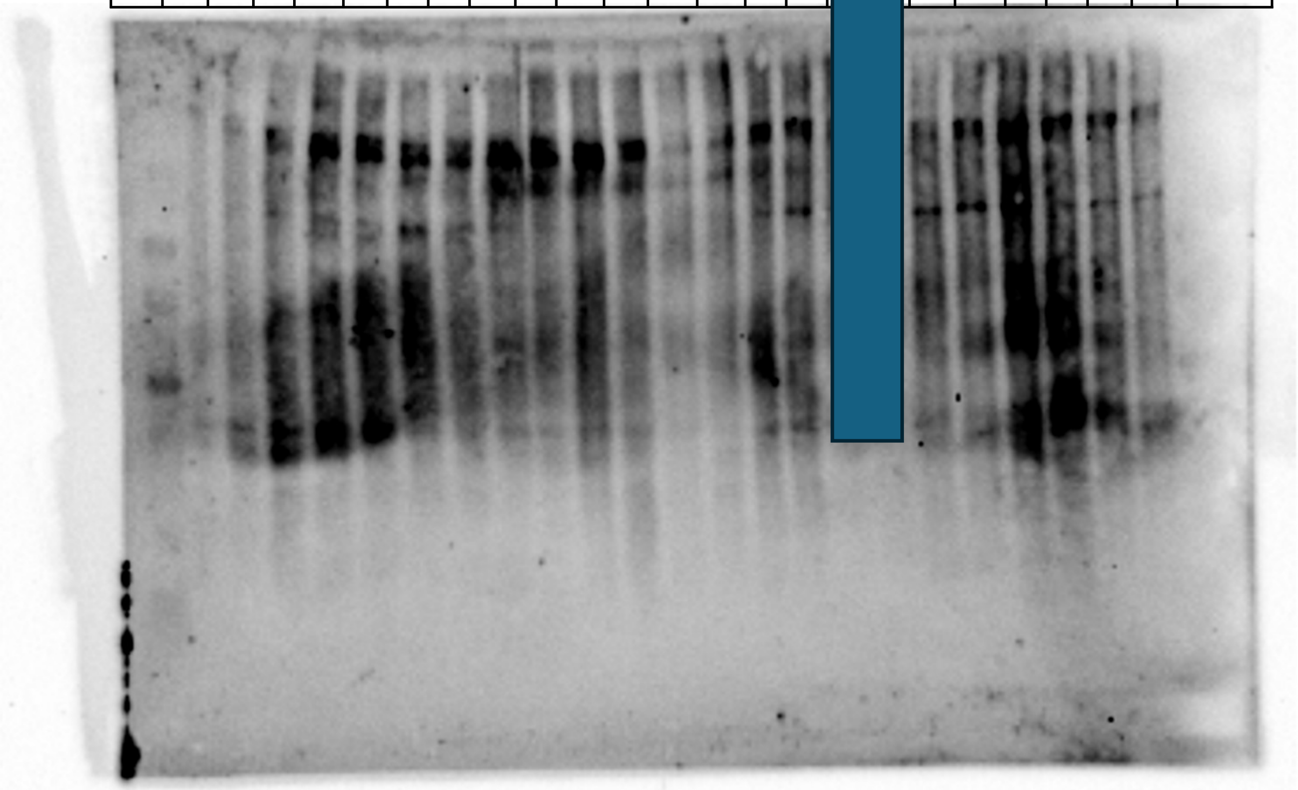

	Calibration curve			H6		O6		H7		O7		H8		O8		[redacted]	O9		H10		H11		
P R	2	4	8	I	I I a	I	I I a	I	I I a	I	I I a	I	II a	I	I I a	[redacted]	I	II a	I	I I a	I	II a	

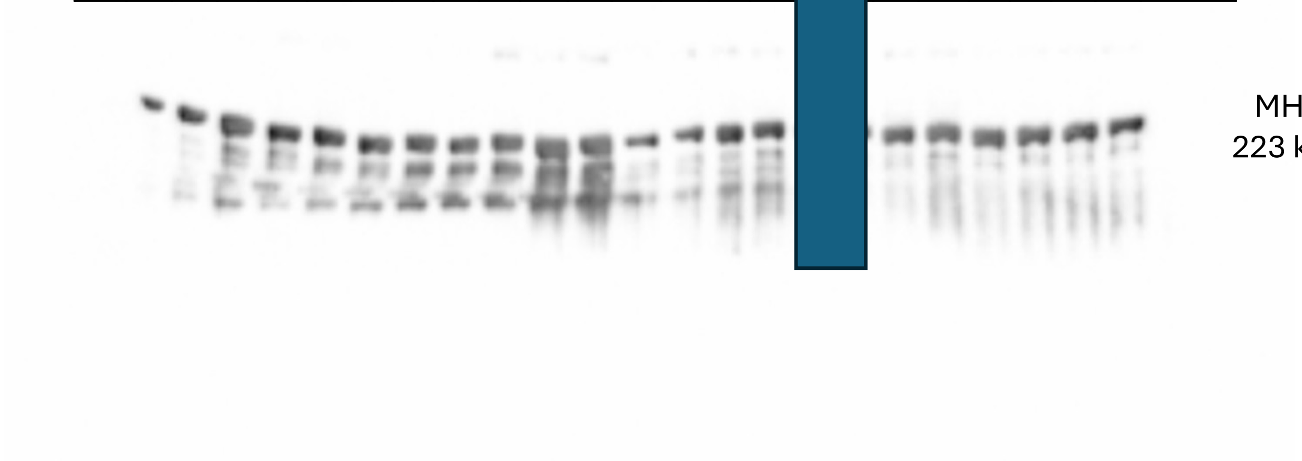

MHC
223 kDa

Ubiquitination whole muscle

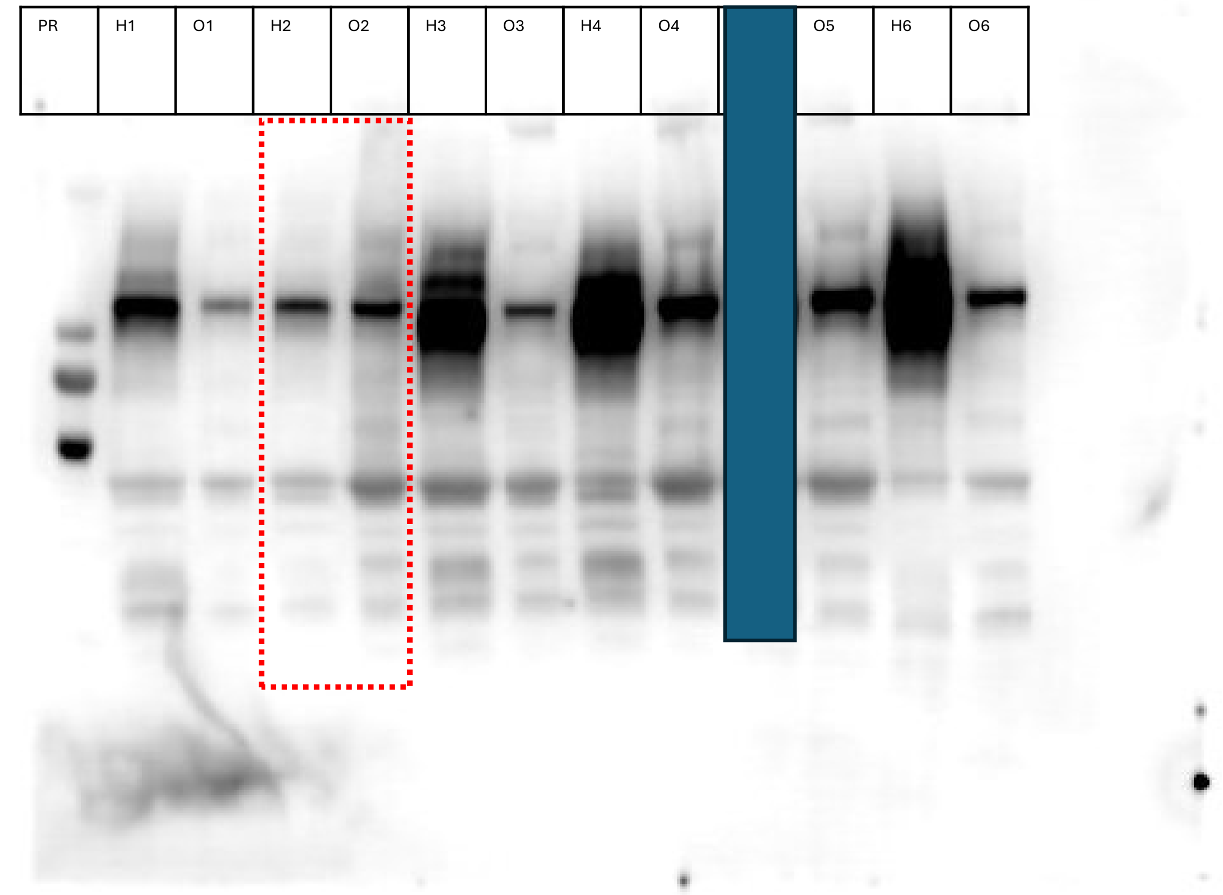

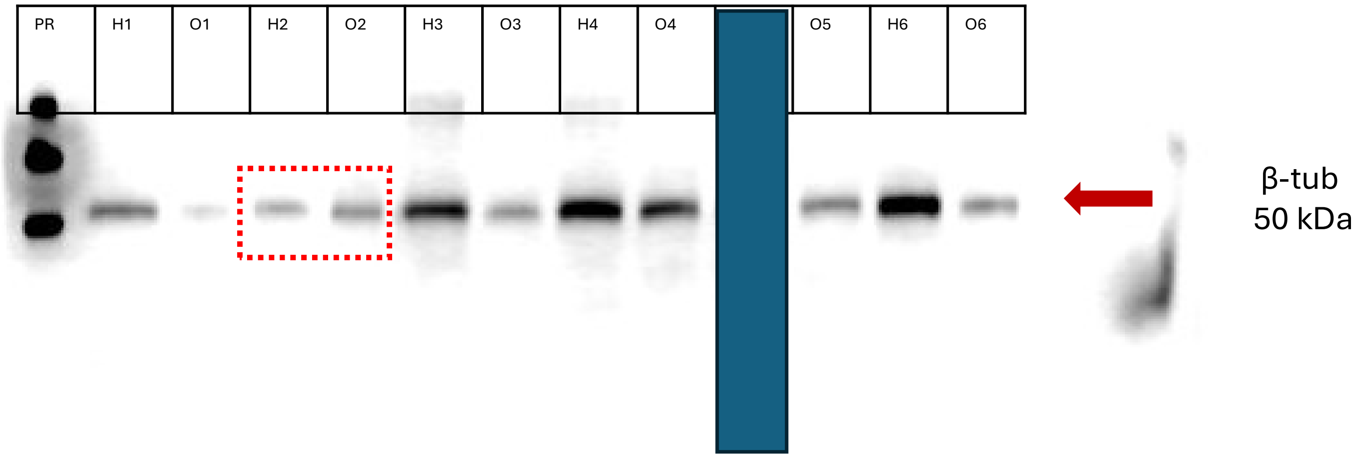

Ubiquitination whole muscle

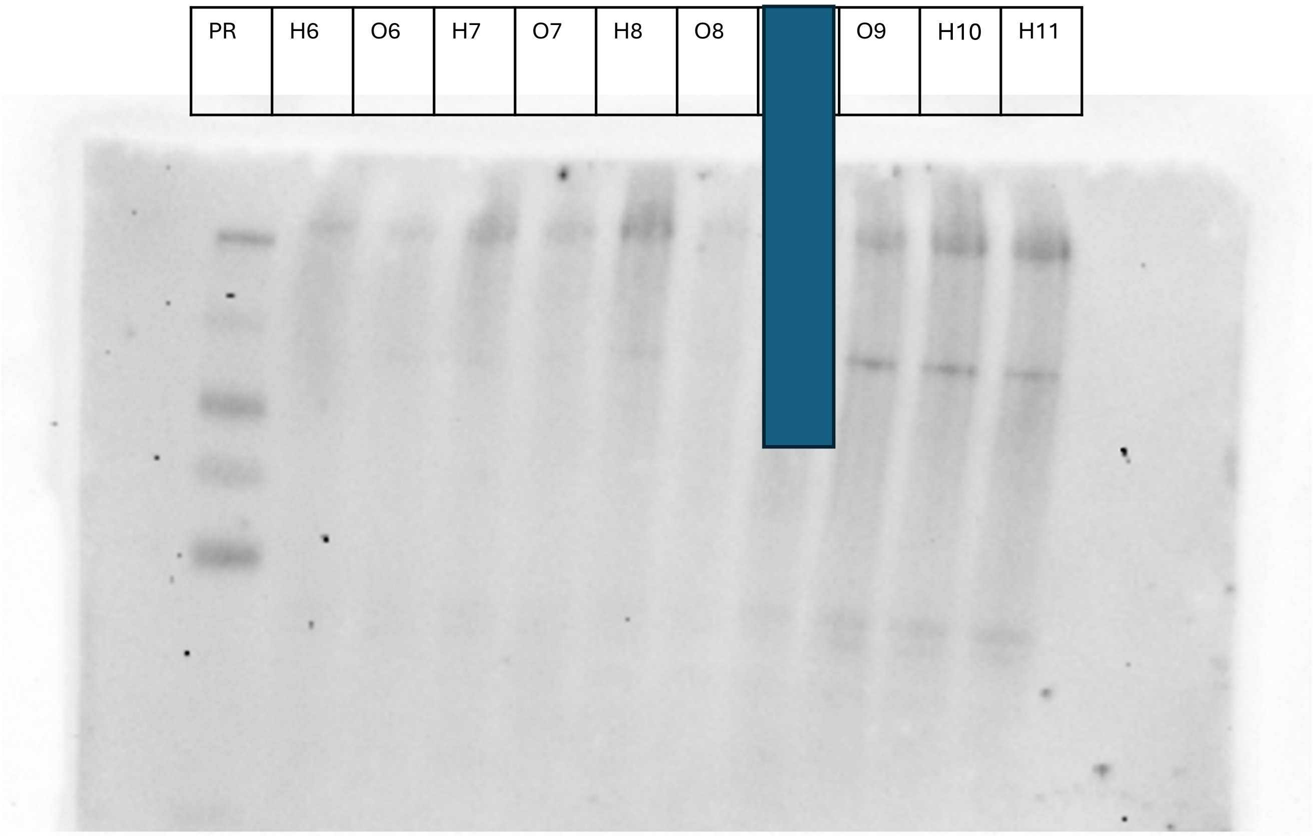

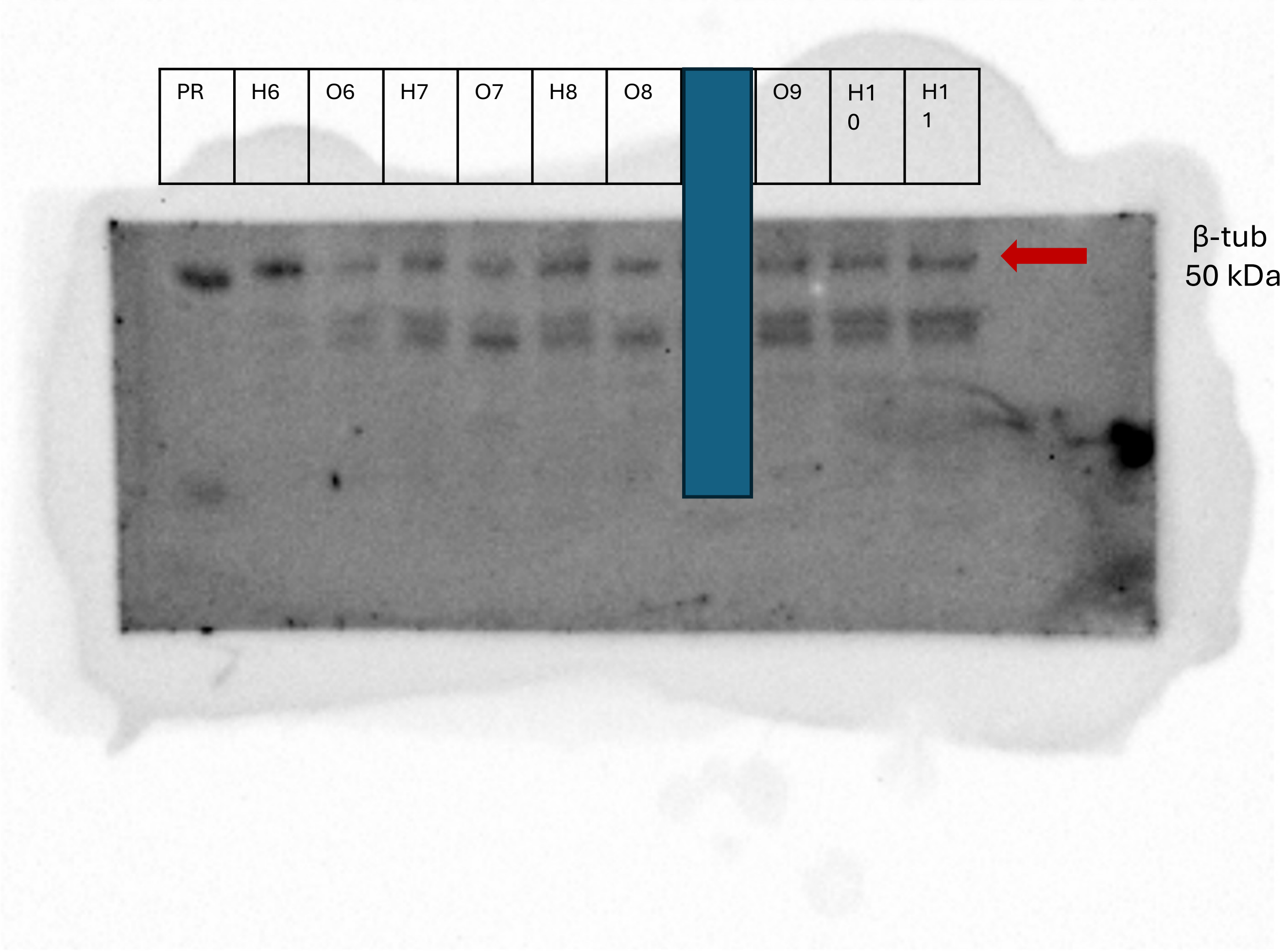

MAPK isolated fibers

Calibration curve (ug)			H1		O1		H2		O2		H3	
2	4	8	I	IIa	I	IIa	I	II a	I	IIa	I	IIa

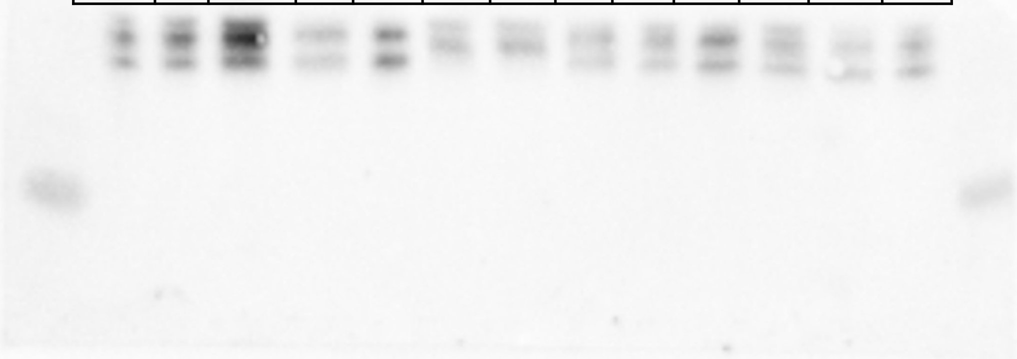

ERK1-2
43-44 kDa

Calibration curve (ug)			H1		O1		H2		O2		H3	
2	4	8	I	IIa	I	IIa	I	II a	I	IIa	I	IIa

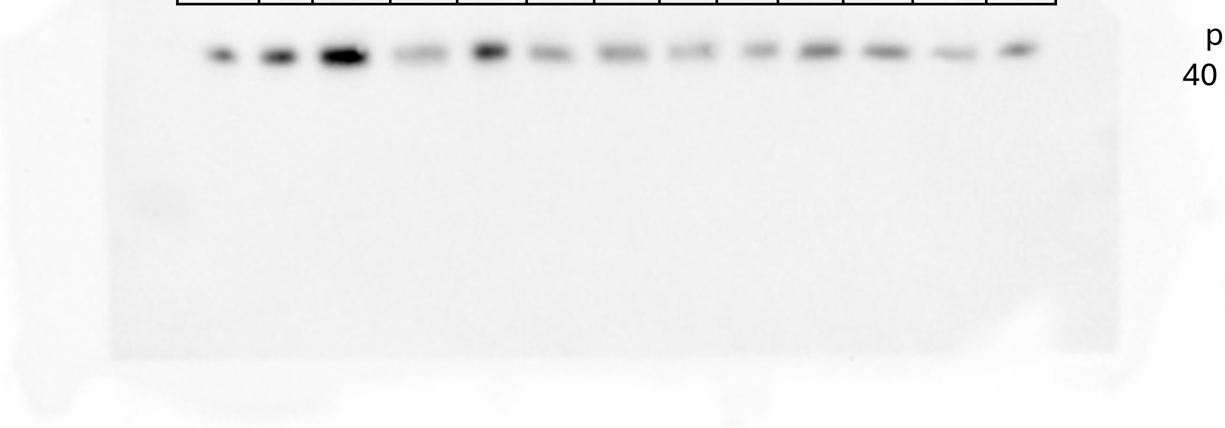

p38
40 kDa

Calibration curve (ug)			H1		O1		H2		O2		H3	
2	4	8	I	IIa	I	IIa	I	II a	I	IIa	I	IIa

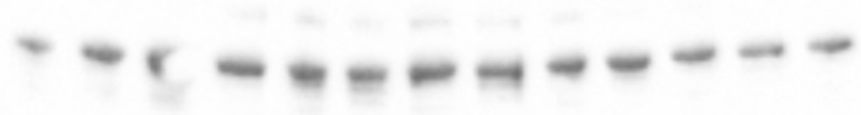

MHC
223 kDa

MAPK isolated fibers

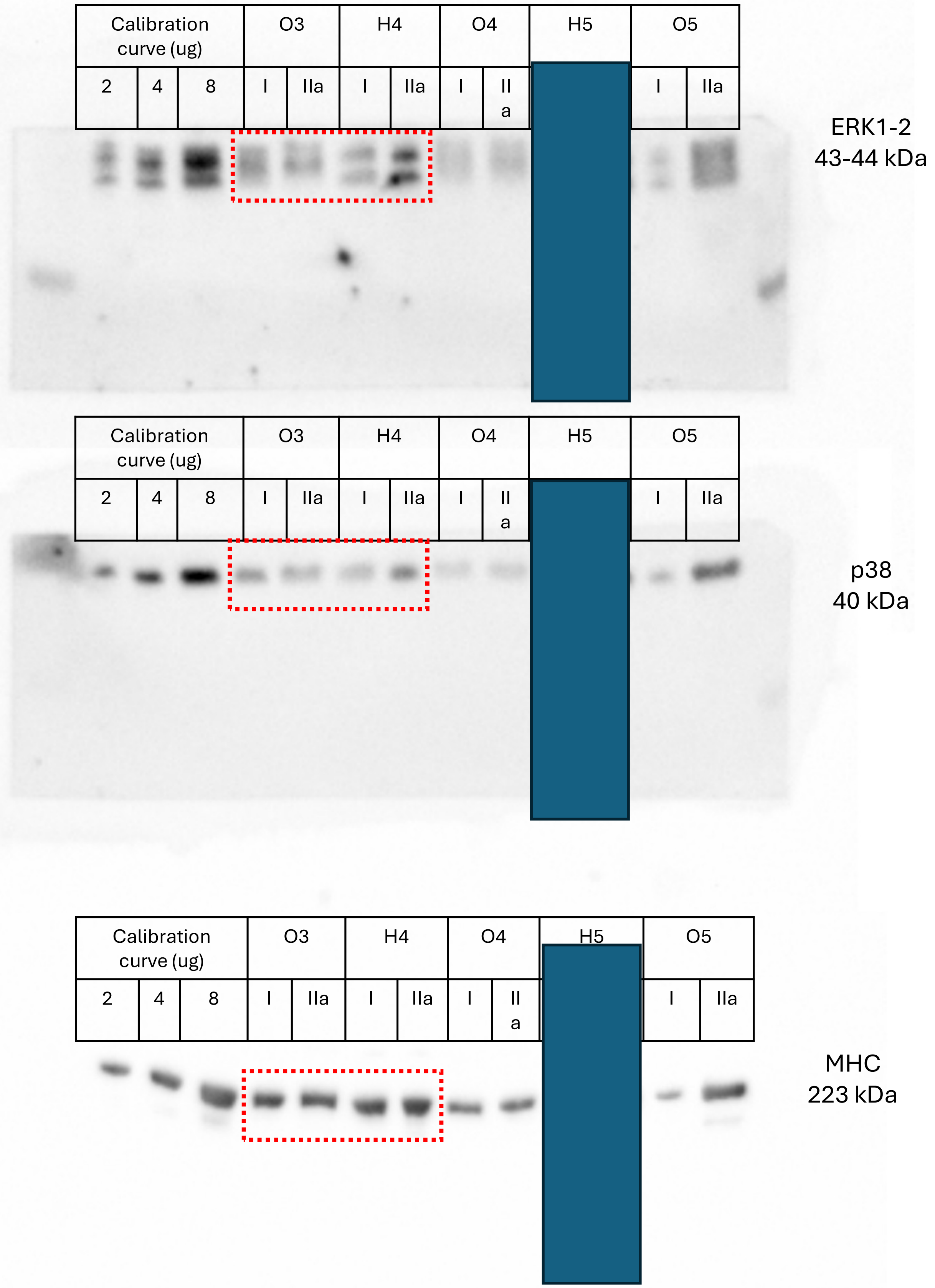

MAPK isolated fibers

Calibration curve (ug)			H6		O6		H7		O7		H8	
2	4	8	I	IIa	I	IIa	I	IIa	I	IIa	I	IIa

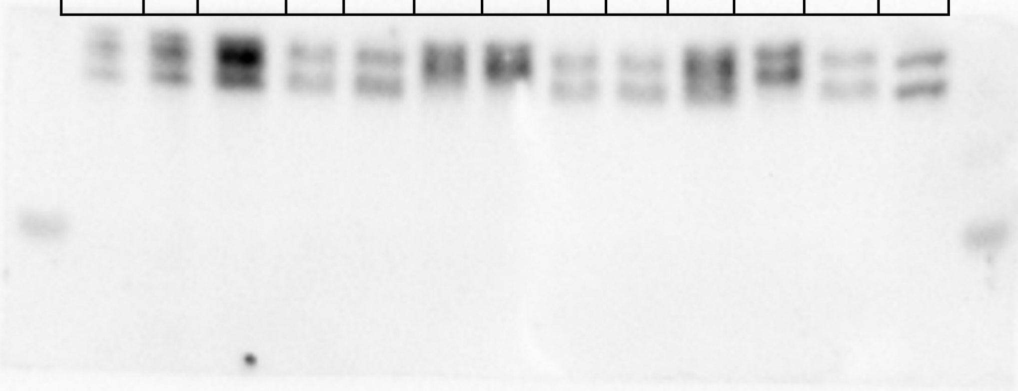

ERK1-2
43-44 kDa

Calibration curve (ug)			H6		O6		H7		O7		H8	
2	4	8	I	IIa	I	IIa	I	IIa	I	IIa	I	IIa

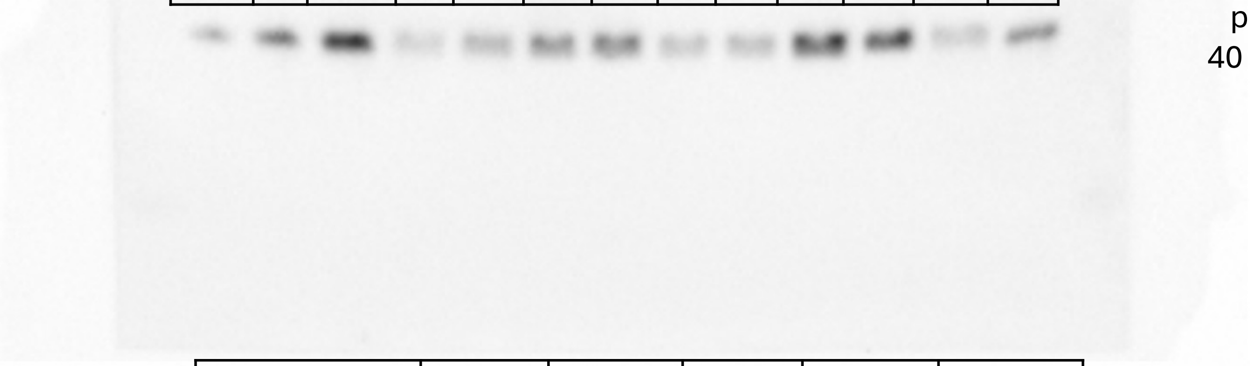

p38
40 kDa

Calibration curve (ug)			H6		O6		H7		O7		H8	
2	4	8	I	IIa	I	IIa	I	IIa	I	IIa	I	IIa

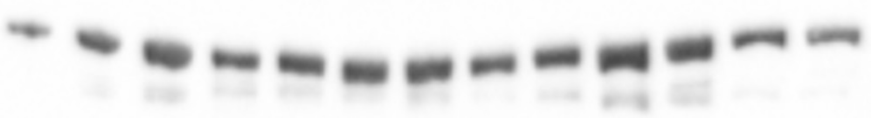

MHC
223 kDa

MAPK isolated fibers

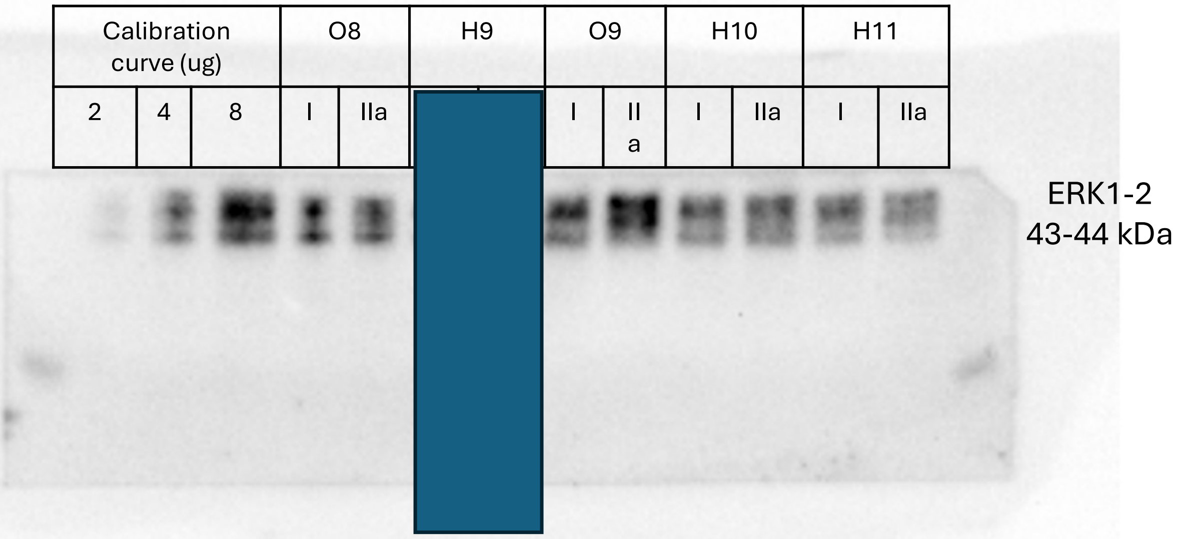

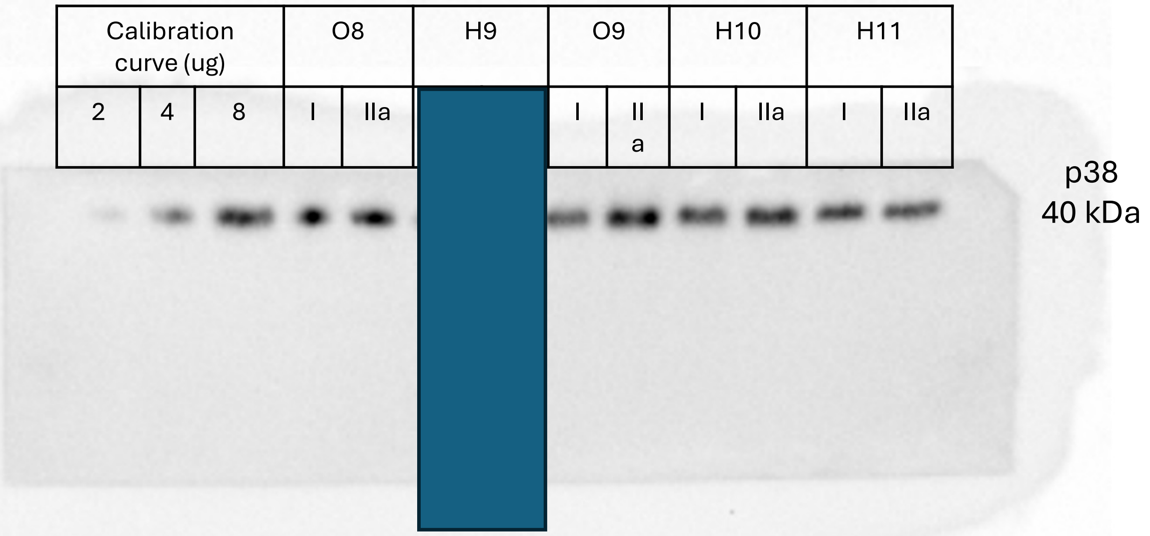

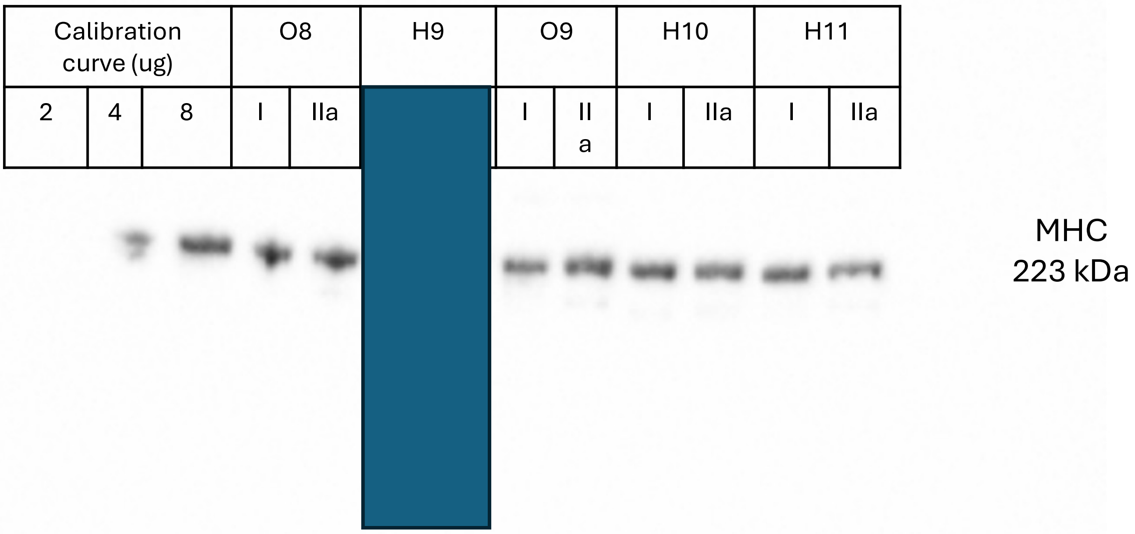

MAPK Whole Muscle

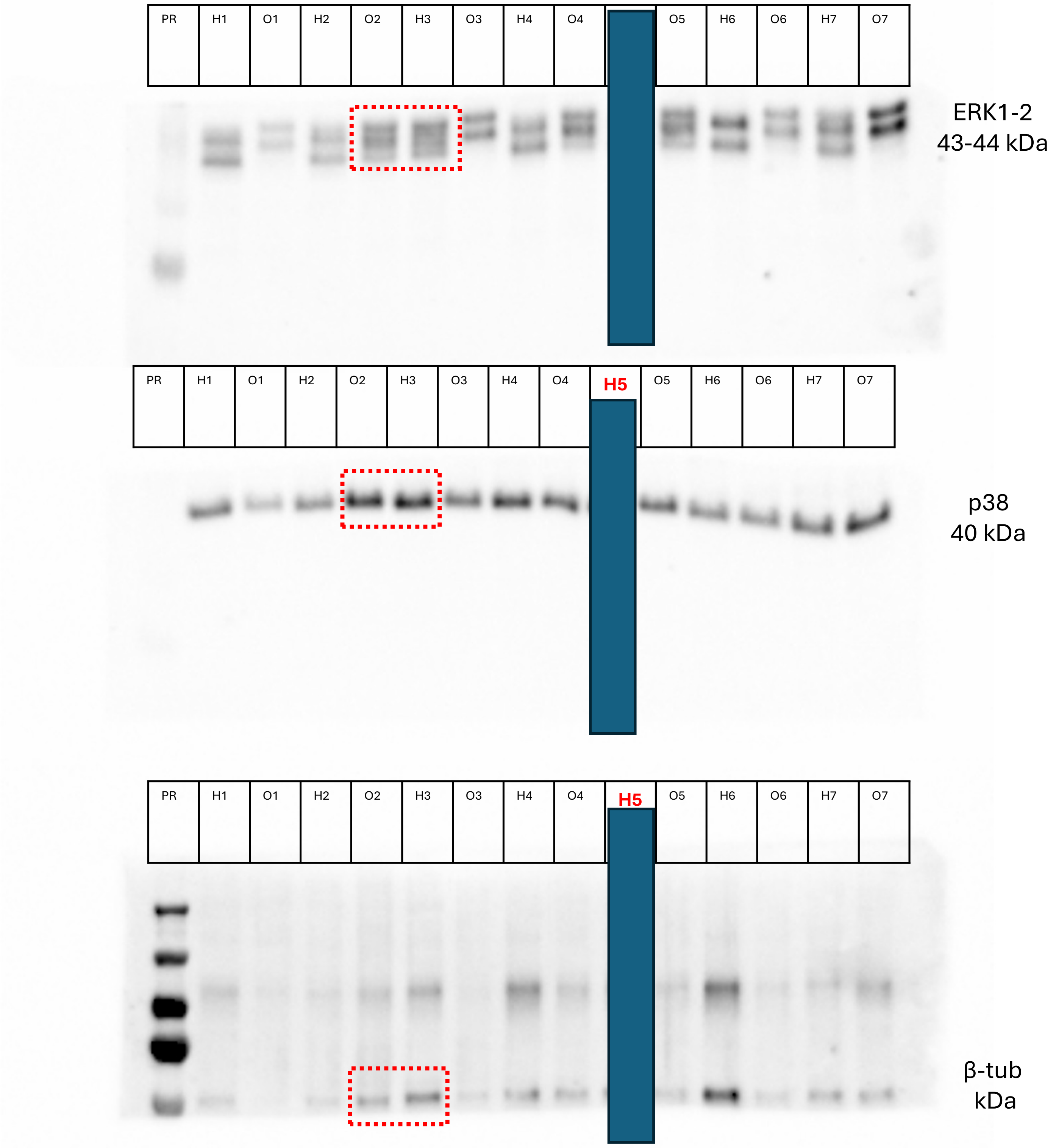

MAPK Whole Muscle

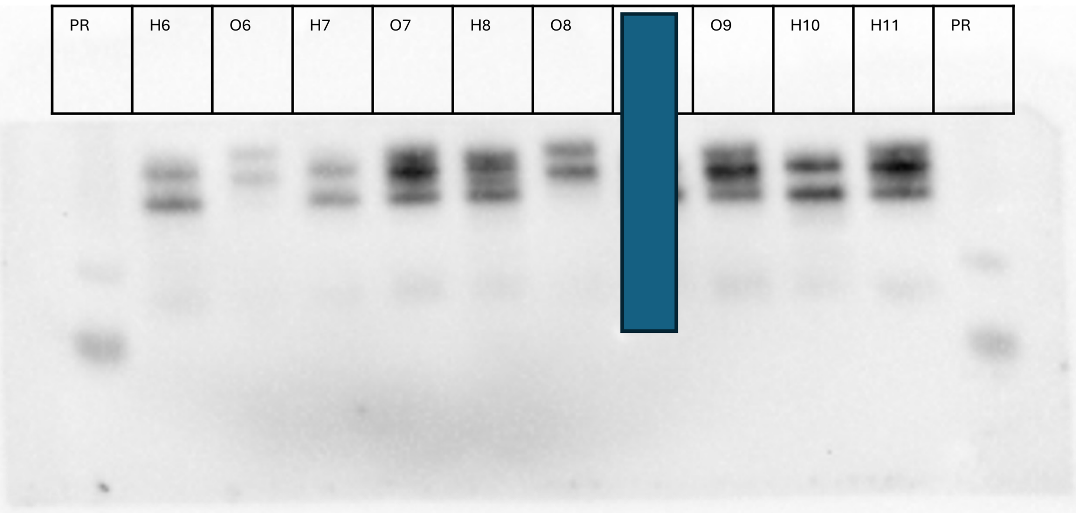

ERK1-2
43-44 kDa

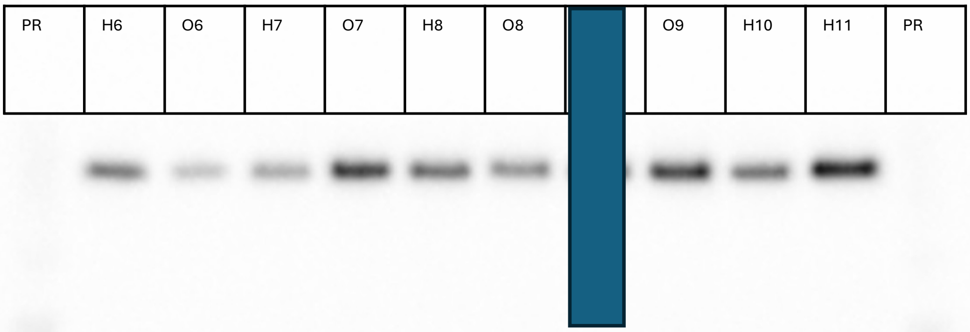

p38
40 kDa

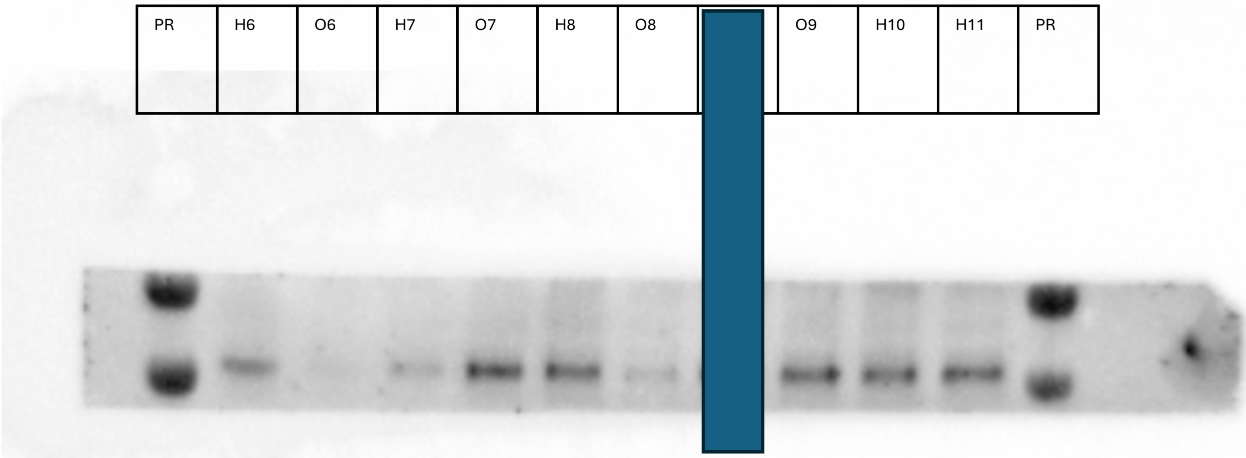

β-tub
kDa

OXPHOS isolated fibers

	H1		O1		H2		O2		Calibration curveUg			
PR	I	IIa	I	IIa	I	IIa	I	IIa	2	4	8	

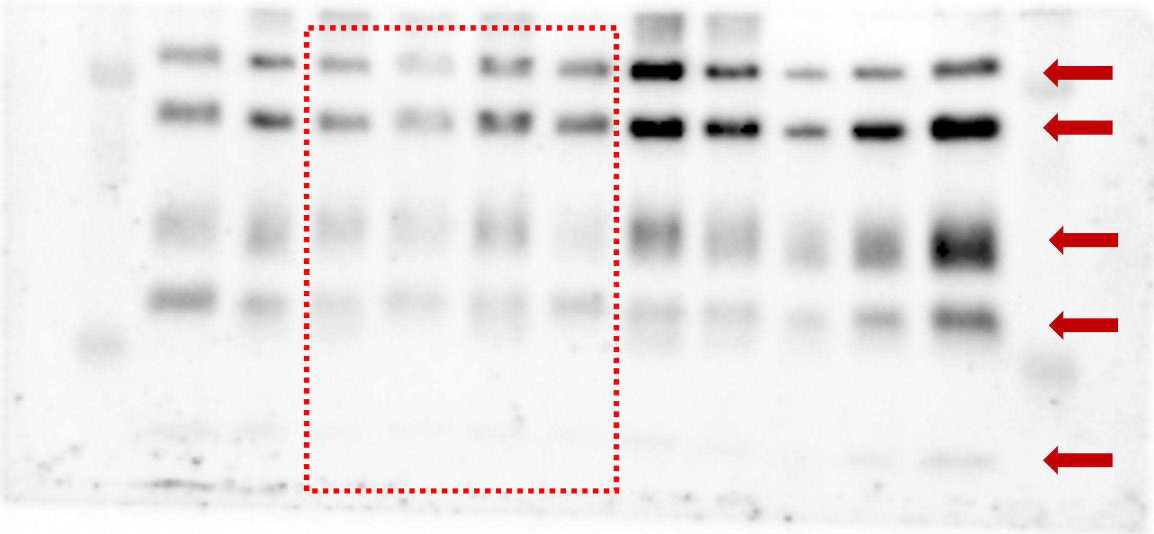

	H1		O1		H2		O2		Calibration curveUg			
PR	I	IIa	I	IIa	I	IIa	I	IIa	2	4	8	

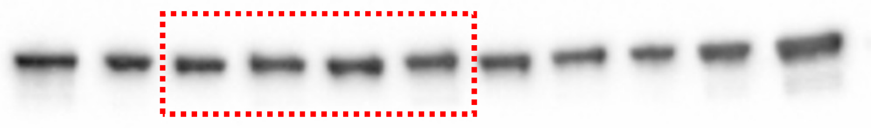

OXPHOS isolated fibers

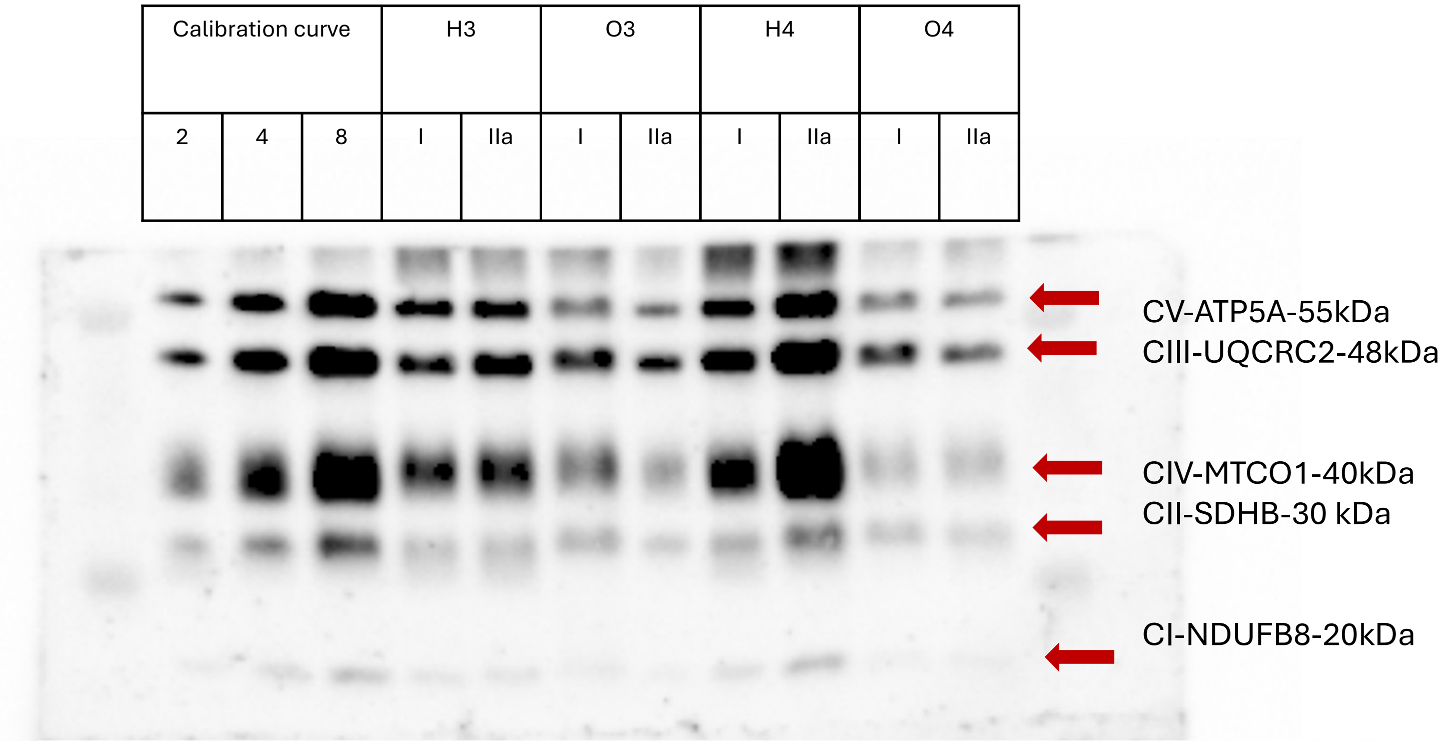

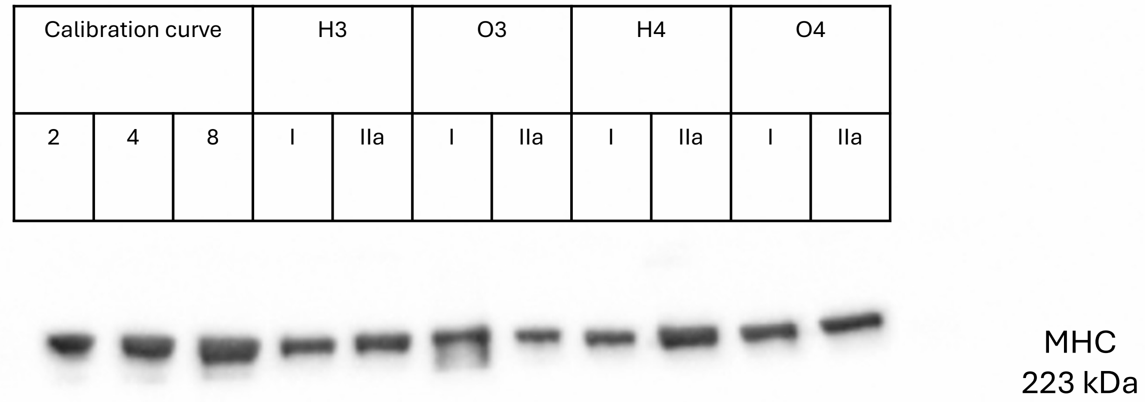

OXPHOS isolated fibers

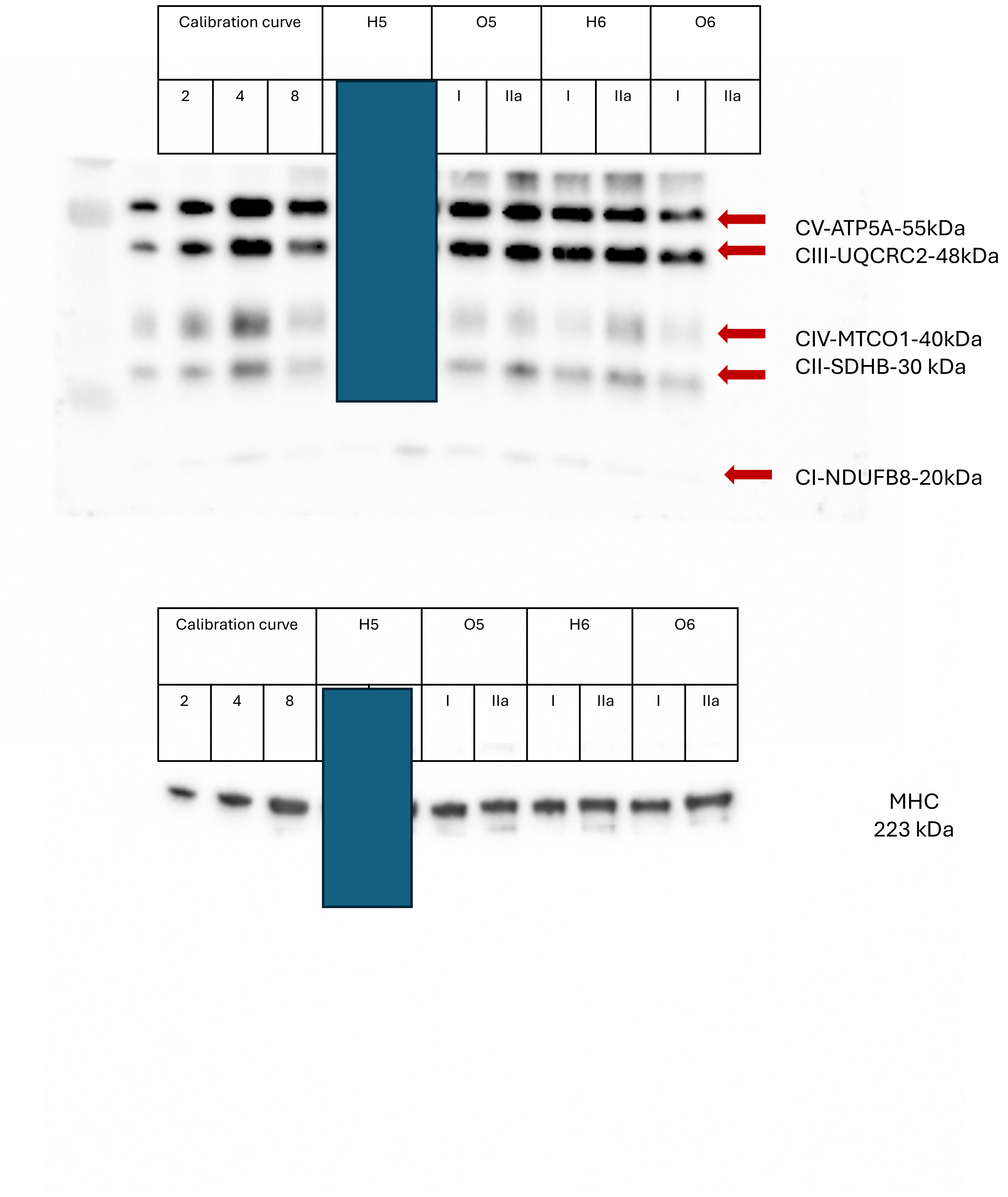

OXPHOS isolated fibers

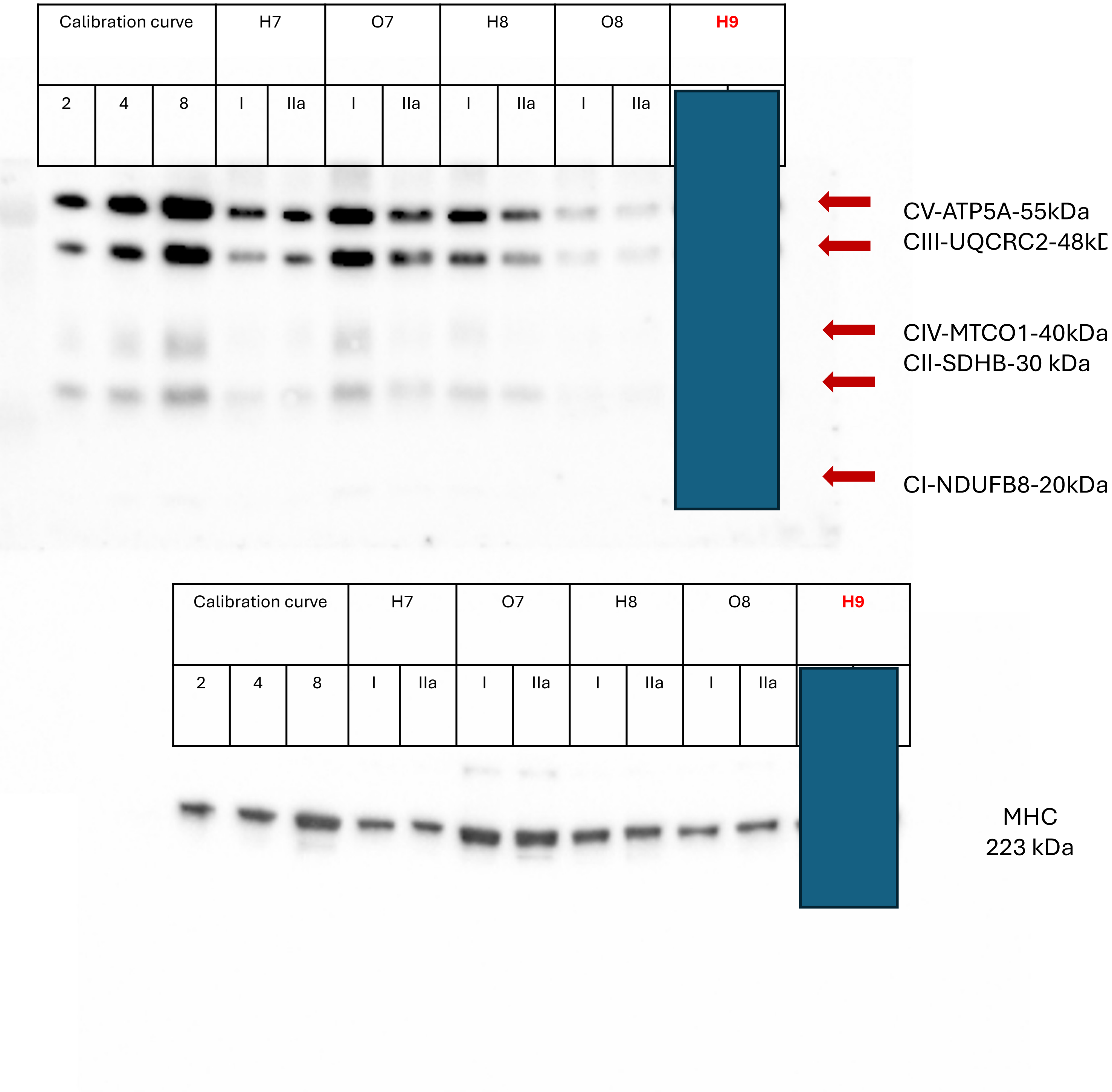

OXPHOS isolated fibers

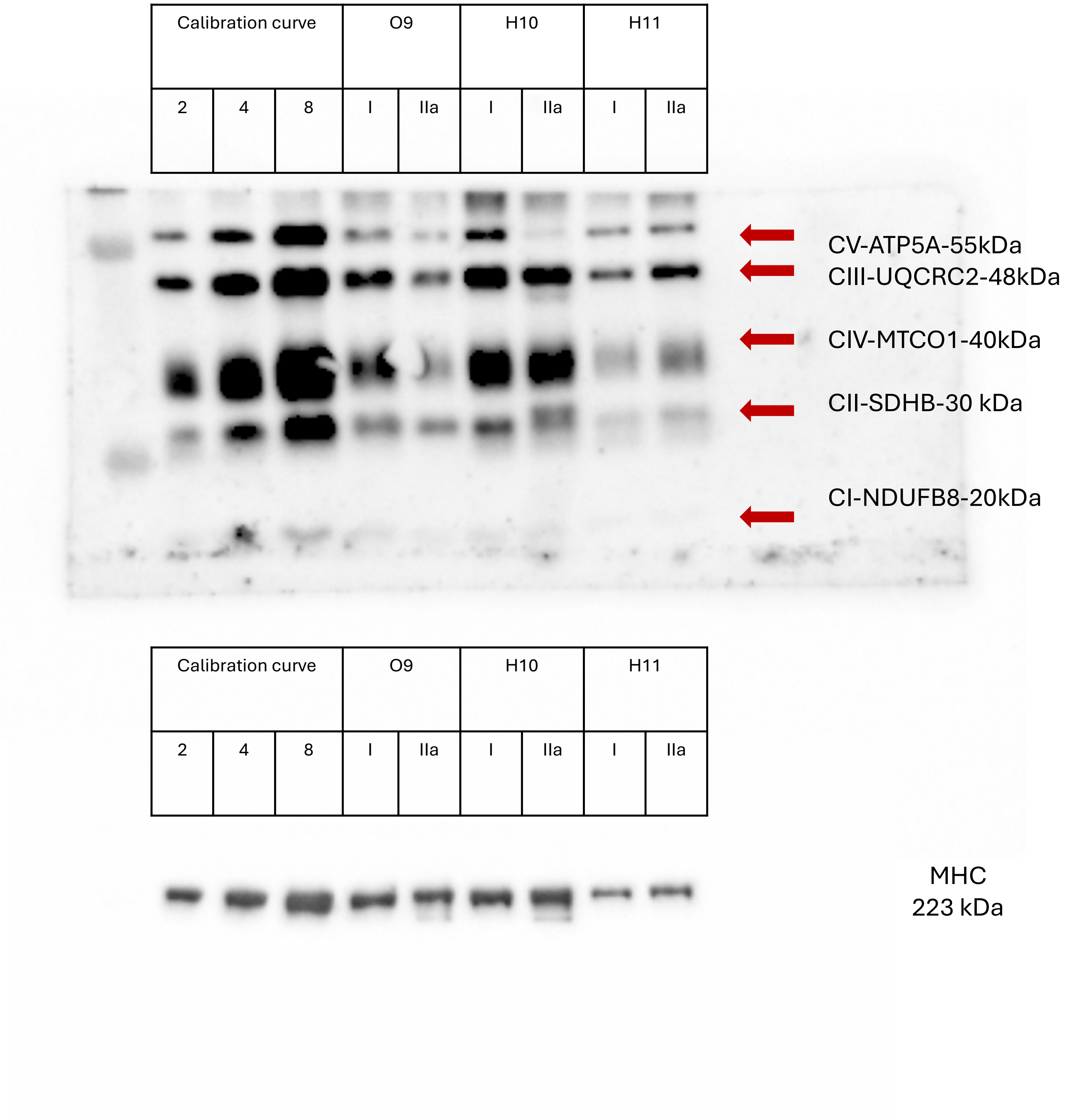

OXPHOS Whole Muscle

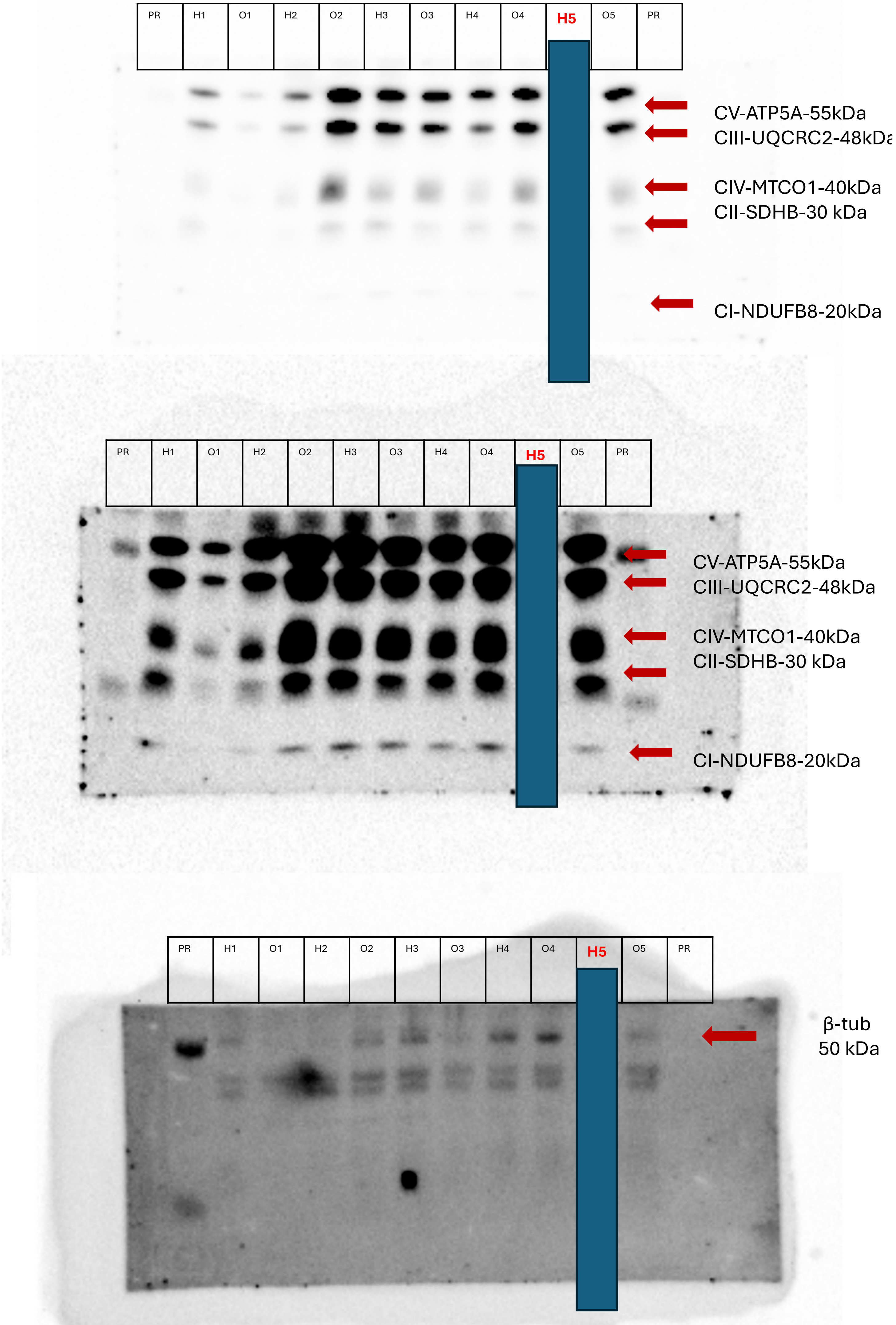

OXPHOS Whole Muscle

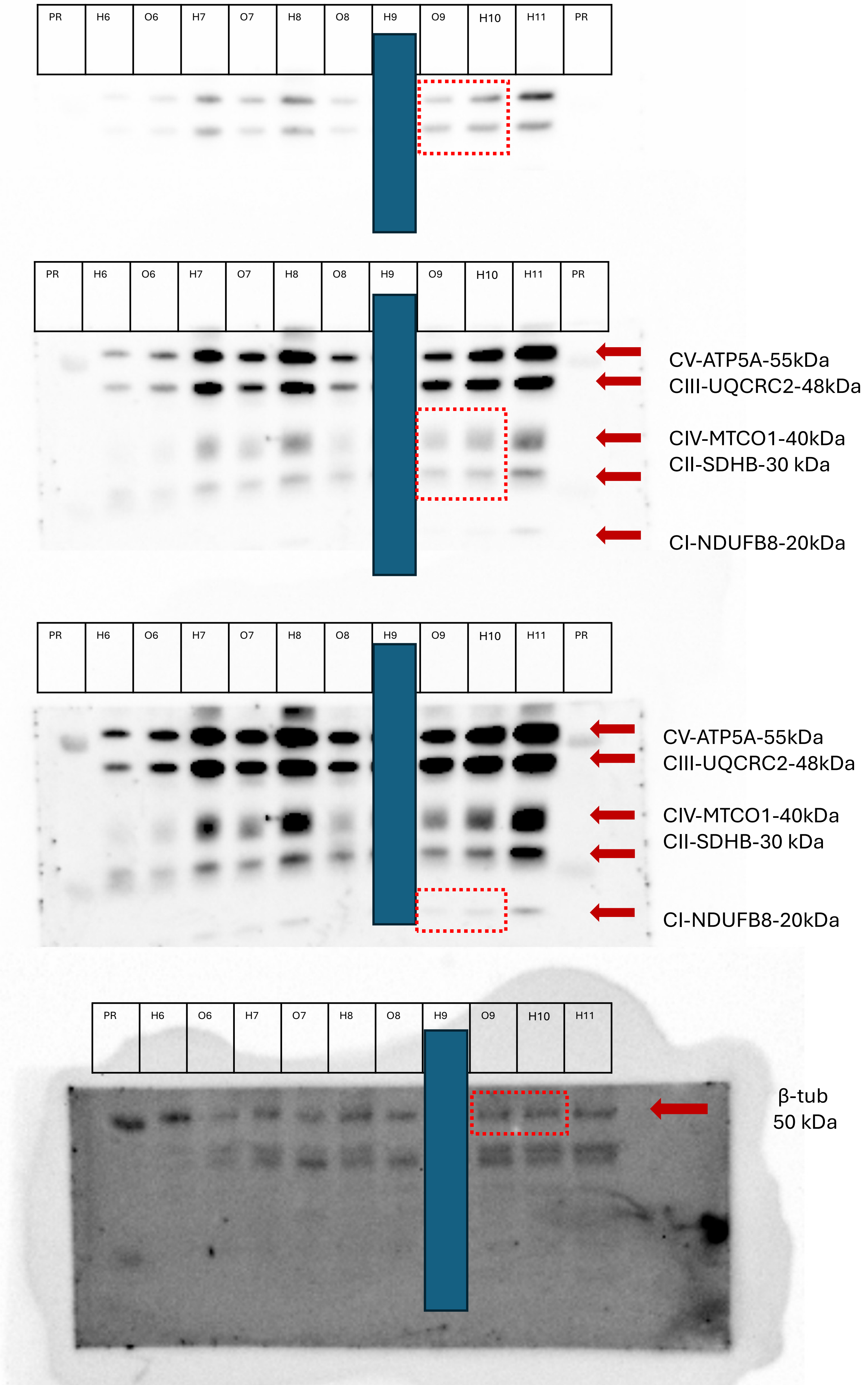

Vimentin

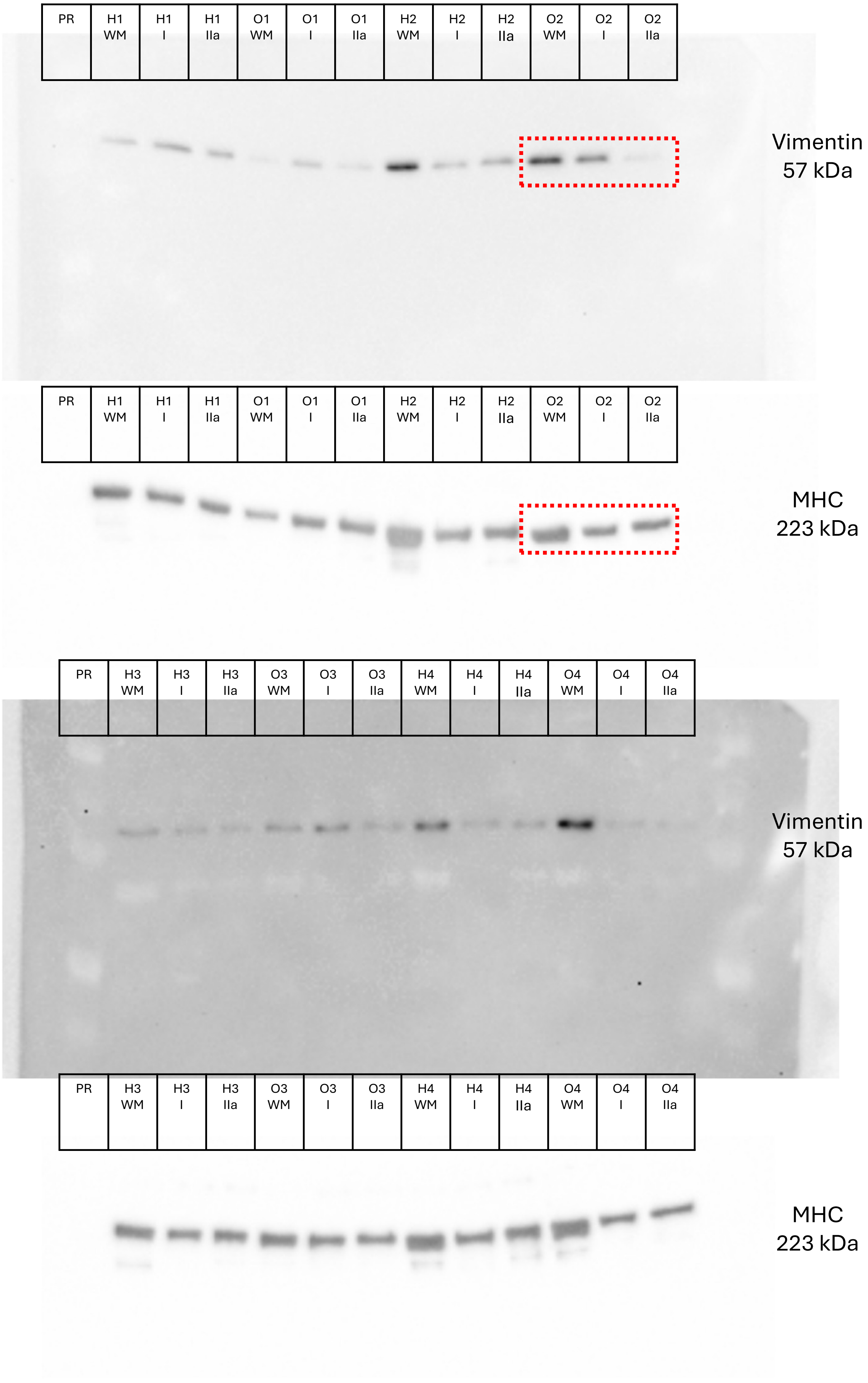
PR
H1 WM
H1 I
H1 IIa
O1 WM
O1 I
O1 IIa
H2 WM
H2 I
H2 IIa
O2 WM
O2 I
O2 IIa
Vimentin 57 kDa
MHC 223 kDa
H3 WM
H3 I
H3 IIa
O3 WM
O3 I
O3 IIa
H4 WM
H4 I
H4 IIa
O4 WM
O4 I
O4 IIa
Vimentin 57 kDa
MHC 223 kDa

Vimentin

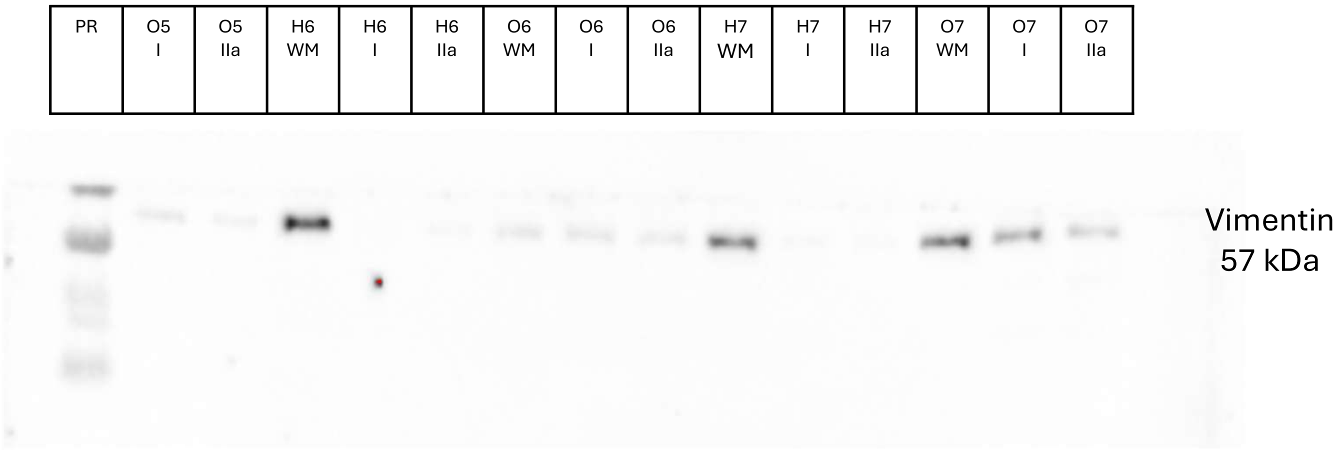
PR
O5 I
O5 IIa
H6 WM
H6 I
H6 IIa
O6 WM
O6 I
O6 IIa
H7 WM
H7 I
H7 IIa
O7 WM
O7 I
O7 IIa
Vimentin
57 kDa

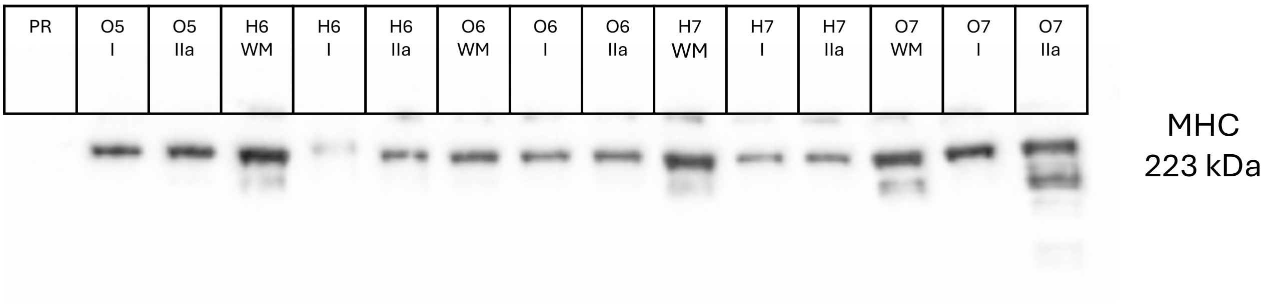
PR
O5 I
O5 IIa
H6 WM
H6 I
H6 IIa
O6 WM
O6 I
O6 IIa
H7 WM
H7 I
H7 IIa
O7 WM
O7 I
O7 IIa
MHC
223 kDa

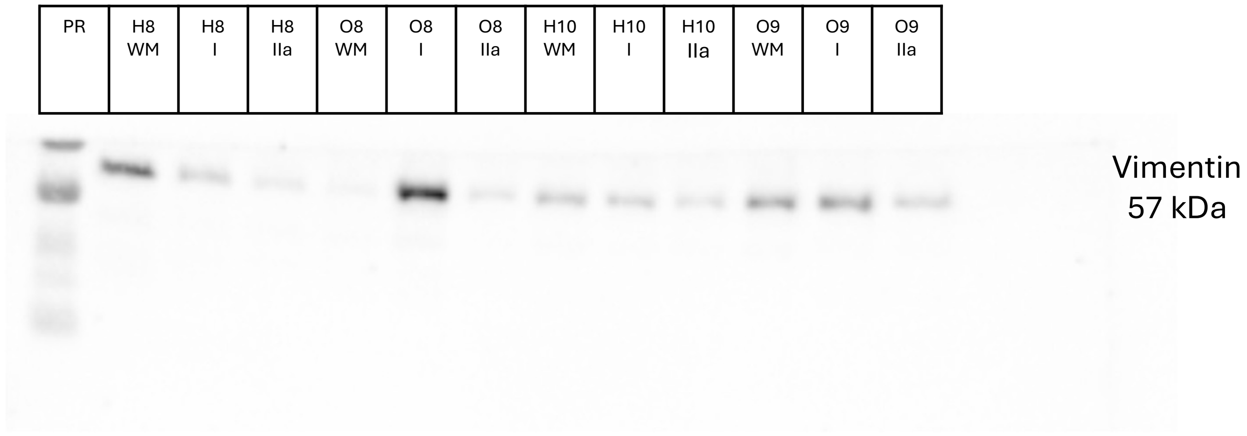
PR
H8 WM
H8 I
H8 IIa
O8 WM
O8 I
O8 IIa
H10 WM
H10 I
H10 IIa
O9 WM
O9 I
O9 IIa
Vimentin
57 kDa

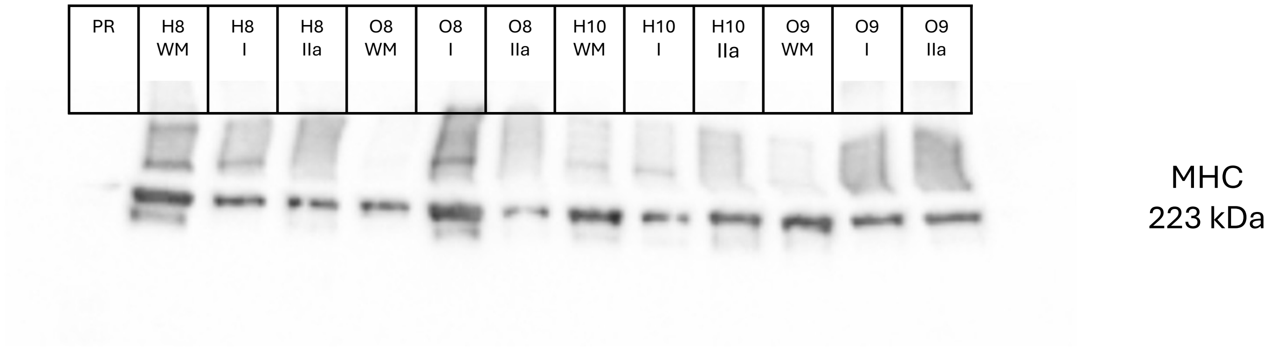
PR
H8 WM
H8 I
H8 IIa
O8 WM
O8 I
O8 IIa
H10 WM
H10 I
H10 IIa
O9 WM
O9 I
O9 IIa
MHC
223 kDa

GFAP

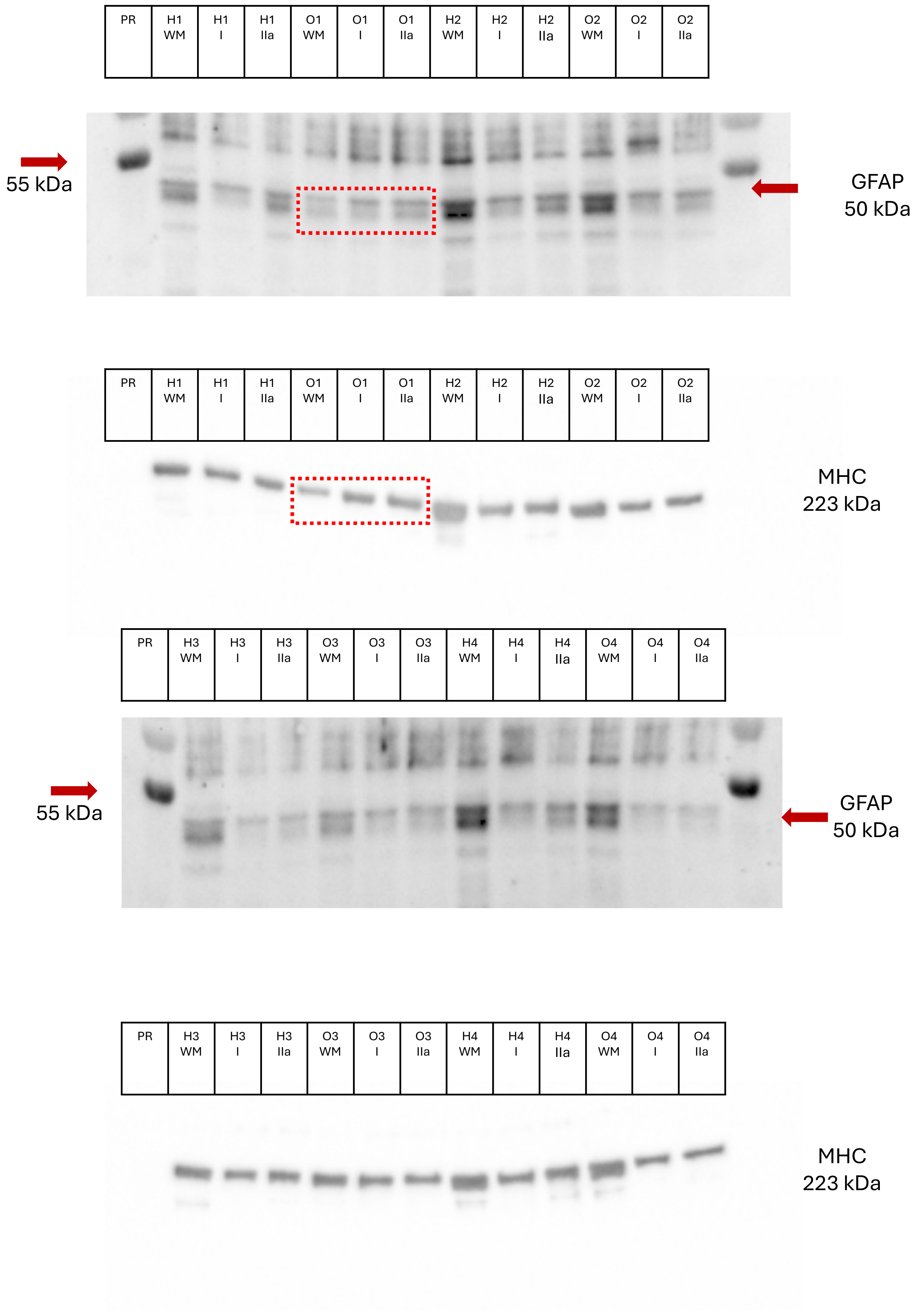
PR
H1 WM
H1 I
H1 IIa
O1 WM
O1 I
O1 IIa
H2 WM
H2 I
H2 IIa
O2 WM
O2 I
O2 IIa
55 kDa
GFAP 50 kDa
PR
H1 WM
H1 I
H1 IIa
O1 WM
O1 I
O1 IIa
H2 WM
H2 I
H2 IIa
O2 WM
O2 I
O2 IIa
MHC 223 kDa
PR
H3 WM
H3 I
H3 IIa
O3 WM
O3 I
O3 IIa
H4 WM
H4 I
H4 IIa
O4 WM
O4 I
O4 IIa
55 kDa
GFAP 50 kDa
PR
H3 WM
H3 I
H3 IIa
O3 WM
O3 I
O3 IIa
H4 WM
H4 I
H4 IIa
O4 WM
O4 I
O4 IIa
MHC 223 kDa

GFAP

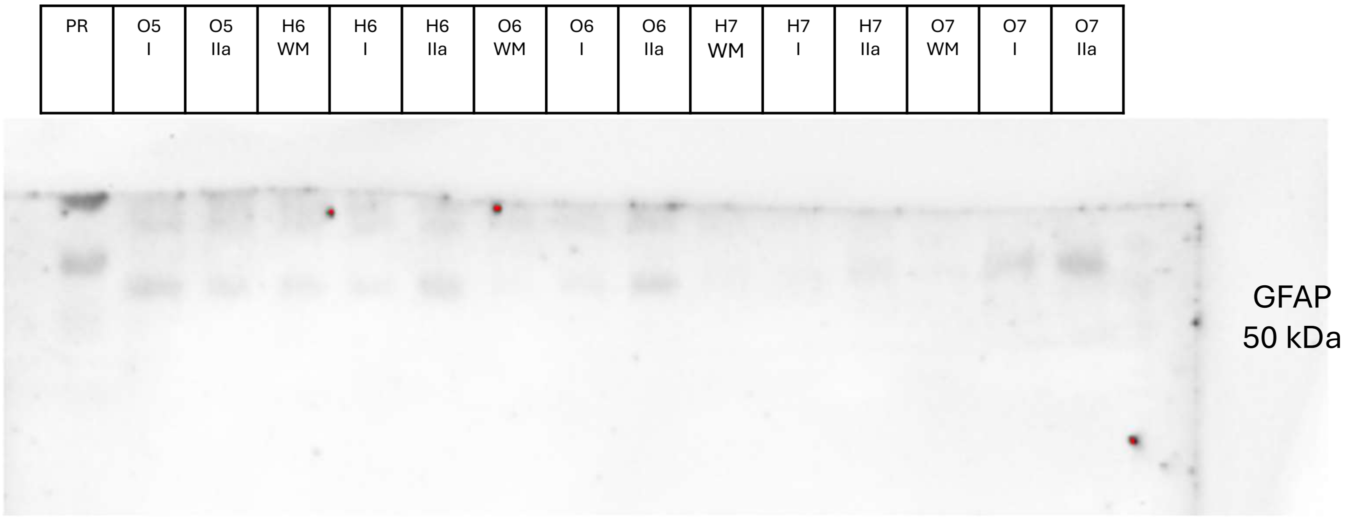
PR
O5 I
O5 IIa
H6 WM
H6 I
H6 IIa
O6 WM
O6 I
O6 IIa
H7 WM
H7 I
H7 IIa
O7 WM
O7 I
O7 IIa
GFAP 50 kDa

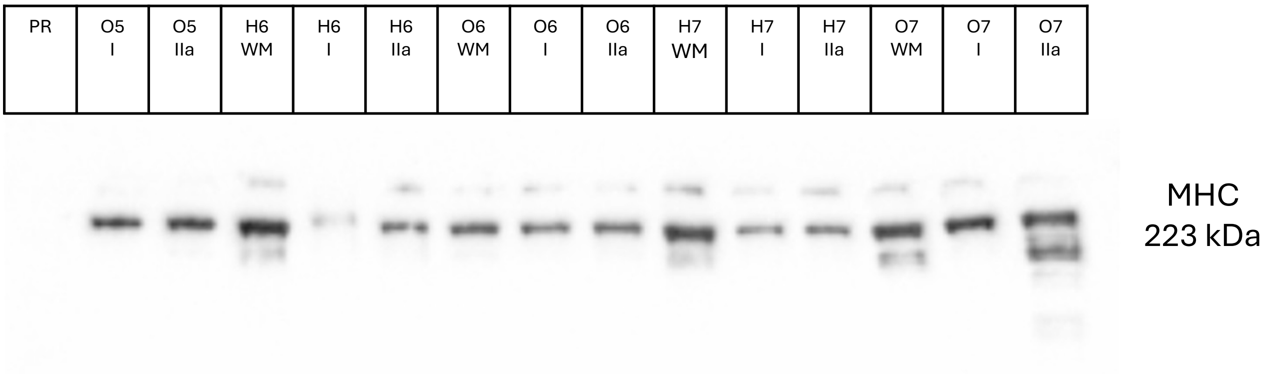
PR
O5 I
O5 IIa
H6 WM
H6 I
H6 IIa
O6 WM
O6 I
O6 IIa
H7 WM
H7 I
H7 IIa
O7 WM
O7 I
O7 IIa
MHC 223 kDa
