# Supplementary material for: Fiber-specific differences in protein content of pathways related to mTORC1 signaling and oxidative metabolism in individuals with obesity
Source: Sci Rep. 2025 Jul 4;15:23839. doi: 10.1038/s41598-025-09169-7 (PMC12229446; doi:10.1038/s41598-025-09169-7)
Supplement: Supplementary file 3 — Supplementary Material 3 [file 41598_2025_9169_MOESM3_ESM.docx]

**Table 2: Primary and secondary antibodies details.**

| Protein | Suppliers | Catalog number | Source/Isotype | Dilution |
| --- | --- | --- | --- | --- |
| **Primary Antibodies used in Western blot** | | | | |
| **mTOR** (25) | Cell Signaling | 2983 | Rabbit IgG | 1:1000 |
| **Akt-1** (26) | Cell Signaling | 2938 | Rabbit IgG |  |
| **P70s6K** (27) | Cell Signaling | 34475 | Rabbit IgG |  |
| **S6RP** (27) | Cell Signaling | 2317 | Mouse IgG |  |
| **Ubiquitin** (28) | Cell Signaling | 43124 | Rabbit IgG |  |
| **ERK1/2** (29) | Cell Signaling | 4695 | Rabbit IgG |  |
| **p38** (30) | Cell Signaling | 8690 | Rabbit IgG |  |
| **Β-tubulin** (32) | Cell Signaling | 2146 | Rabbit IgG |  |
| **GFAP** (36) | Cell Signaling | 3670 | Mouse IgG |  |
| **Vimentin** (37) | Cell Signaling | 5741 | Rabbit IgG |  |
| **OXPHOS** (31) | Invitrogen | 45-8099 | Mouse IgG | 1:2000 |
| **Primary Antibodies used in Dot blot** | | | | |
| **MCH** (33) | DSHB | MF20-s | Mouse-IgG | 1:4000 |
| **MCH I** (24) | DSHB | A4840-s | Mouse-IgM |  |
| **MCH IIa** (24) | DSHB | A474-s | Mouse-IgG |  |
| **Secondary Antibodies** | | | | |
| **Anti-mouse IgG-HRP** | Cell Signaling | 7076 |  | 1:2000 |
| **Anti-mouse IgM-HRP** | Invitrogen | 62-6820 |  |  |
| **Anti-rabbit IgG-HRP** | Cell Signaling | 7074 |  |  |
